# COVID-19 vaccination induces cross-neutralisation of sarbecoviruses related to SARS-CoV-2

**DOI:** 10.1038/s41541-026-01469-x

**Published:** 2026-07-01

**Authors:** Grace E. West, Rebecca B. Morse, Benjamin L. Sievers, Adam Abdullahi, Kimia Kamelian, Mark Tsz Kin Cheng, The CITIID-NIHR BioResource COVID-19 Collaboration, John R. Bradley, Stephen Baker, Barbara Graves, Hannah Stark, Sabine Hein, Ingrid Scholtes, Daniela Caputo, Anne Elmer, Emma Le Gresley, Nathalie Kingston, Patrick Chinnery, Daniel Cooper, Gordon Dougan, Ian Goodfellow, Nathalie Kingston, Paul J. Lehner, Paul A. Lyons, Nicholas J. Matheson, Caroline Saunders, Kenneth G. C. Smith, Charlotte Summers, James Thaventhiran, M. Estee Torok, Mark R. Toshner, Michael P. Weekes, Gisele Alvio, Sharon Baker, Areti Bermperi, Karen Brookes, Isabel Cruz, Ranalie de Jesus, Katie Dempsey, Giovanni Di Stephano, Jason Domingo, Sarah Hewitt, Heather Jones, Sherly Jose, Jenny Kourampa, Caroline McMahon, Vivien Mendoza, Charmain Ocaya, Ciro Pasquale, Marlyn Perales, Carla Ribeiro, Vargas, Bensi Vergese, Laura Watson, Jieniean Worsley, Julie-Ann Zerrudo, Laura Bergamashi, Gräf, Kelvin Hunter, Federica Mescia, John Allison, Heather Biggs, Helen Butcher, Matt Chandler, Debbie Clapham-Riley, Eleanor Dewhurst, Christian Fernandez, Anita Furlong, Jennifer Gray, Tasmin Ivers, Rachel Linger, Mary Kasanicki, Rebecca King, Sarah Meloy, Alexei Moulton, Francesca Muldoon, Nigel Ovington, Sofia Papadia, Christopher J. Penkett, Isabel Phelan, Venkatesh Ranganath, Roxana Paraschiv, Abigail Sage, Jennifer Sambrook, Katherine Schon, Kathleen E. Stirrups, Paul Townsend, Neil Walker, Jennifer Webster, Petra Polgarova, Sarah L. Caddy, Laura G. Caller, Yasmin Chaudhry, Martin D. Curran, Theresa Feltwell, Stewart Fuller, Iliana Georgana, Grant Hall, William L. Hamilton, Myra Hosmillo, Charlotte J. Houldcroft, Rhys Izuagbe, Aminu S. Jahun, Fahad A. Khokhar, Anna G. Kovalenko, Luke W. Meredith, Surendra Parmar, Malte L. Pinckert, Anna Yakovleva, Emily C. Horner, Lucy Booth, Alexander Ferreira, Rebecca Boston, Robert Hughes, Juan Carlos Yam Puc, Nonantzin Beristain-Covarrubias, Maria Rust, Thevinya Gurugama, Lihinya Gurugama, Thomas Mulroney, Sarah Spencer, Zhaleh Hosseini, Kate Williamson, Douglas G. Stewart, Divya Diamond, Mazharul Altaf, Lourdes Ceron-Gutierrez, Olga Sokolova, David L. Robertson, Spyros Lytras, Rainer Döffinger, Sam J. Wilson, Suzannah J. Rihn, Ravindra K. Gupta

**Affiliations:** 1 University of Cambridge, Cambridge, UK; 2 Division of Systems Virology, Department of Microbiology and Immunology, The Institute of Medical Science, The University of Tokyo, Tokyo, Japan; 3 MRC-University of Glasgow Centre for Virus Research, The University of Glasgow, Glasgow, UK; 4 HKJC Global Health Institute, Hong Kong SAR, China; 5 NIHR BioResource, Cambridge University Hospitals NHS Foundation Trust, Cambridge Biomedical Campus, Cambridge, UK; 6 Cambridge Clinical Research Centre, Addenbrooke’s Hospital, Cambridge University Hospitals NHS Foundation Trust, Cambridge, UK; 7 Department of Clinical Neurosciences, School of Clinical Medicine, University of Cambridge, Cambridge Biomedical Campus, Cambridge, UK; 8 Medical Research Council Mitochondrial Biology Unit, Cambridge Biomedical Campus, Cambridge, UK; 9 Cambridge University Hospitals NHS Foundation Trust, Cambridge Biomedical Campus, Cambridge, UK; 10 Global and Tropical Health Division, Menzies School of Heath Research and Charles Darwin University, Darwin, NT Australia; 11 Department of Public Health and Primary Care, School of Clinical Medicine, University of Cambridge, Cambridge Biomedical Campus, Cambridge, UK; 12 Division of Virology, Department of Pathology, University of Cambridge, Cambridge, UK; 13 Department of Infectious Diseases, Addenbrooke’s Hospital, Cambridge University NHS Hospitals Foundation Trust, Cambridge, UK; 14 NHS Blood and Transplant, Cambridge, UK; 15 Department of Renal Medicine, Addenbrooke’s Hospital, Cambridge University Hospitals NHS Foundation Trust, Cambridge, UK; 16 Intensive Care Unit, Addenbrooke’s Hospital, Cambridge University Hospitals NHS Foundation Trust, Cambridge Biomedical Campus, Cambridge, UK; 17 Heart and Lung Research Institute, Cambridge Biomedical Campus, Cambridge, UK; 18 MRC Toxicology Unit, Gleeson Building, Tennis Court Road, Cambridge, UK; 19 Department of Microbiology, Addenbrooke’s Hospital, Cambridge University NHS Hospitals Foundation Trust, Cambridge, UK; 20 Royal Papworth Hospital NHS Foundation Trust, Cambridge, UK; 21 Cambridge Institute for Medical Research, Biomedical Campus, Hills Rd, Cambridge, UK; 22 Intensive Care Unit, Royal Papworth Hospital NHS Foundation Trust, Cambridge, UK; 23 Africa Health Research Institute, Durban, South Africa; 24 Clinical Genetics, Addenbrooke’s Hospital, Cambridge University Hospitals NHS Foundation Trust, Cambridge, UK; 25 The Francis Crick Institute, London, UK; 26 Public Health England, Clinical Microbiology and Public Health Laboratory, Cambridge, UK

**Keywords:** Immunology, Vaccines, RNA vaccines

## Abstract

The combined threats of future sarbecovirus zoonosis and continually emerging SARS-CoV-2 VOCs highlight the need to assess the breadth of existing SARS-CoV-2 vaccine-mediated protection. Here, we investigate a cohort of older individuals who received four COVID-19 vaccine doses, for potential cross-neutralisation against lentiviral particles bearing spikes from either Omicron VOCs or other sarbecoviruses. Despite recent fourth bivalent mRNA vaccine doses (encoding SARS-CoV-2 Wu-1 and Omicron spikes), neutralisation of Omicron lineage VOCs was reduced compared to Wu-1, consistent with an imprinted immune response. Similarly, particles bearing either SARS-CoV-1 or a SARS-CoV-1-related bat sarbecovirus spike were neutralised less efficiently than Wu-1. Unexpectedly, however, we observed that particles with spikes from two animal SARS-CoV-2-related viruses, BANAL-20-52 from bats and a pangolin CoV, were significantly more sensitive to serum neutralising antibodies than SARS-CoV-2 Wu-1 itself. These surprising findings suggest that vaccine-mediated adaptive immunity may provide efficient cross-neutralisation and potential protection against certain animal sarbecoviruses.

## Introduction

The importance of zoonotic virus transmission in global public health was highlighted in 2019 when SARS-CoV-2, a coronavirus similar to several circulating coronaviruses in bats, emerged in humans in Wuhan, China^1^. This marked the third major coronavirus outbreak in humans over the previous two decades, following the SARS and MERS outbreaks of 2003 and 2012, respectively. During the subsequent COVID-19 pandemic, the swift development of monovalent vaccines based on the host receptor-binding spike protein from the first SARS-CoV-2 sequence isolated in Wuhan in 2019 (SARS-CoV-2 Wu-1)^1^ was instrumental in controlling transmission and protecting at-risk populations, which included adults aged over 65 and immunocompromised people^2^.

Despite the generally high initial efficacy of these vaccines^3,4^, older and immunocompromised individuals had lower neutralising antibody titres^5,6^, and across immunocompetent adults, antibody titres were observed to wane more rapidly within six months^7,8^. Thus, booster regimens were introduced; a third dose of monovalent vaccine as a booster established effective neutralising titres against the original SARS-CoV-2 Wu-1 strain^3,4^, as well as reasonable, short-lived neutralisation against emerging variants of concern (VOCs)^9^. VOCs are characterised by nucleic acid substitutions throughout the viral genome^10^, however, there has been specific interest in substitutions in the spike glycoprotein, which mediates cell entry, and in particular the spike’s receptor binding domain (RBD), which interacts with the host cell receptor ACE2 (angiotensin-converting enzyme 2). Non-synonymous substitutions within the RBD, such as L452R and E484Q, are associated with increased transmissibility, infection, and immune escape, and can thereby facilitate breakthrough infections post-vaccination^11–13^. Moreover, the emergence of the Omicron lineage VOCs dramatically increased infection rates, particularly affecting those aged 65 and above^14,15^. Since the recognition of VOC emergence as an ongoing threat to vaccination efforts, bivalent and variant-specific vaccines have been deployed to optimise neutralisation of new VOCs that are now recognised as emerging in older and immunosuppressed individuals^16,17^.

SARS-CoV, hereafter referred to as SARS-CoV-1, and SARS-CoV-2, along with many dozens of other SARS-related coronaviruses (SARSr-CoVs), belong to the *Sarbecovirus* subgenus within the Betacoronavirus genus of the *Coronaviridae* family. The majority of sarbecoviruses that have been identified to date have been found in bats belonging to the *Rhinolophus* genus (horseshoe bats). More specifically, the species *Rhinolophus sinicus* is thought to be a putative reservoir for progenitors of SARS-CoV-1, and possibly also SARS-CoV-2^1,18,19^, and various other SARSr-CoVs have also been isolated from this species (Supplementary Table 1)^20–23^. Although sarbecoviruses have been identified in diverse regions globally, most have been sampled from *Rhinolophus* bats in southern China and south east Asia, which often overlap with regions of high human population^24^. This has raised questions about whether different sarbecoviruses from bats or other wild species might have further potential to enter humans and cause future epidemics or pandemics, particularly as previous work has suggested that humans living in these regions are routinely exposed to and infected by other bat SARSr-CoVs^25^. Of additional concern is the fact that since SARS-CoV-2 is now endemic in the human population, coinfection of humans could lead to recombination, a common occurrence amongst coronaviruses generally, and also specifically within bat SARSr-CoVs, which could generate antigenically novel variants^18^.

There is thus great interest in identifying both the individual animal viruses that may be more likely to enter humans, and what features might enable them to do so. This includes, but is not limited to, the ability to enter human cells using a host receptor, ability to use host machinery to replicate, and the capacity to overcome any innate or adaptive immune barriers. With regard to animal sarbecoviruses, a key determinant of their cross-species transmission potential is likely to be the capacity of their spikes to engage human ACE2, the primary entry receptor for SARS-CoV-1 and SARS-CoV-2, or to utilise human transmembrane protease, serine 2 (TMPRSS2) to enter cells^1,26,27^. In addition to the RBD, other features of the spike may determine its ability to bind to host receptors, including the presence of sites that allow the spike to be cleaved by host proteases. For example, a furin cleavage site is present in the spike of SARS-CoV-2 but has not been found in any sarbecoviruses from bats or pangolins, an important feature required for the identification of a direct progenitor^28,29^.

Nevertheless, a number of animal sarbecoviruses have now been reported to bind or utilise human ACE2/TMPRSS2 entry pathways^1,26,27,30^, and there is no evidence that any significant adaptation was required prior to spillover^31,32^. Moreover, while some bat sarbecoviruses, such as RaTG13 from *Rhinolophus affinis*^1^, share a relatively high overall genome similarity to SARS-CoV-2, they can have varying levels of similarity in different intragenic regions due to recombination within their ancestry. RaTG13, for example, is more divergent in the region of the viral spike that binds to host receptors^33^, whereas other sarbecoviruses have nearly identical receptor-binding regions. In particular, BANAL-20-52 and BANAL-20-236, which were identified in *Rhinolophus* samples from northern Laos, differ from SARS-CoV-2 by only one or two amino acid residues in the receptor-binding motif (RBM), respectively^28^. In addition, some sarbecoviruses isolated from pangolins have spikes that share close genetic similarity to the spike of SARS-CoV-2, and appear to be able to enter human cells, raising questions as to whether pangolins could be conceivable intermediate hosts in the spillover of SARS-CoV-2 from bats to humans^34–36^.

Given that some animal sarbecoviruses appear poised to potentially spillover into humans once more, it is crucial to understand whether existing COVID-19 vaccine-mediated immunity might offer protection against these sarbecoviruses. This may be particularly important for individuals at increased risk of severe disease and will also enable the development of forward-thinking vaccination strategies. In this study focusing on older adults in the UK, a clinically relevant group that is at a higher risk of severe COVID-19 outcomes, we investigate whether COVID-19 vaccines are able to cross-neutralise the entry of pseudotyped lentiviral particles bearing spikes from Omicron VOCs and a selection of SARS-CoV-1 and SARS-CoV-2 related bat and pangolin sarbecoviruses that can utilise ACE2, some of which have fewer substitutions within the receptor-binding regions of their spikes than VOCs relative to SARS-CoV-2 Wu-1. We thus aim to examine the role that existing vaccines may have in future epidemic or pandemic mitigation.

## Results

### Neutralisation sensitivity of BA.2-derived VOCs following a fourth vaccine dose in older adults

**Fig. 1:**
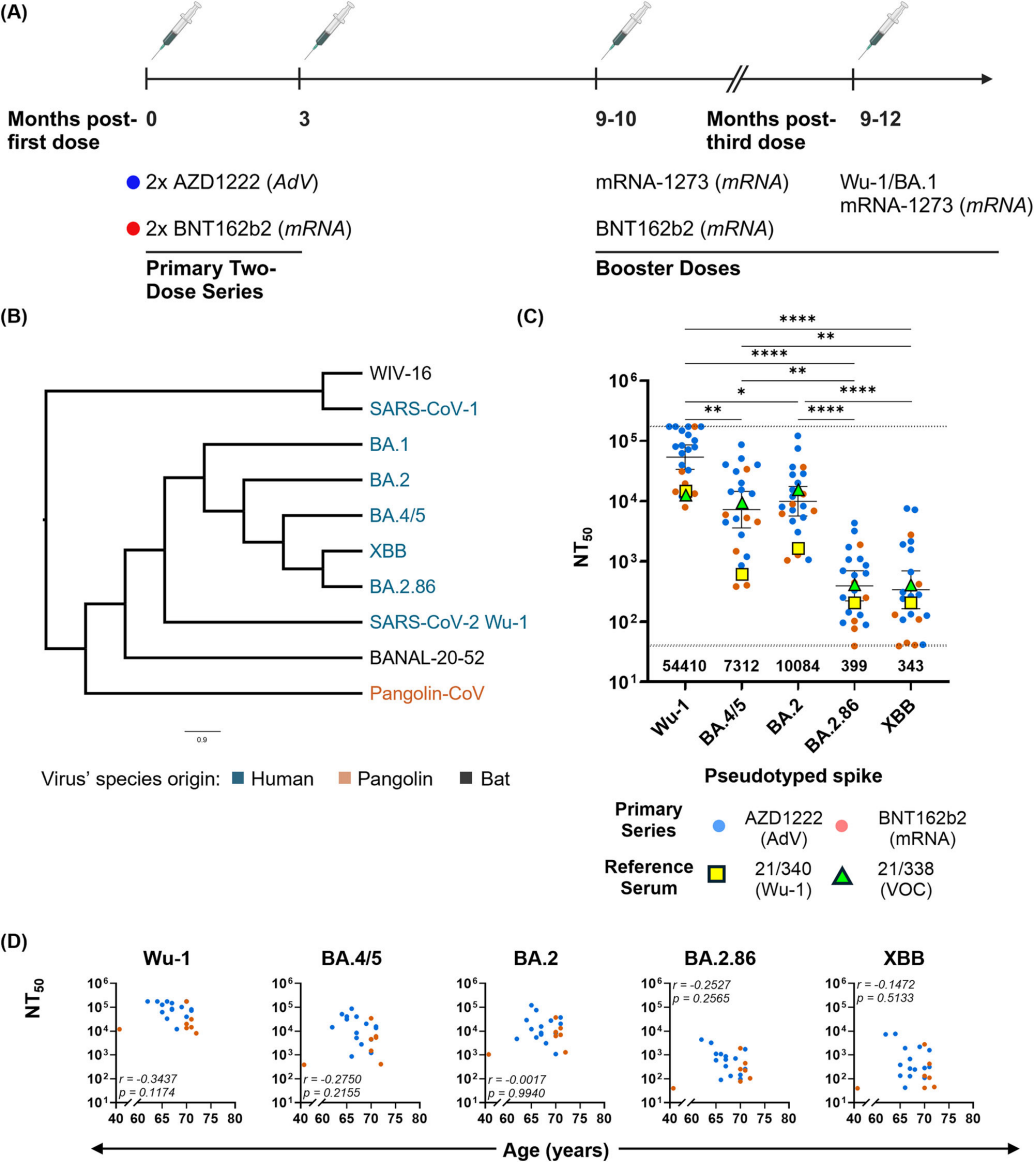
Multi-dose vaccine schedules in a cohort of older individuals with mixed infection histories induce strong humoral responses against SARS-CoV-2 Wu-1 and early Omicron lineages. **A** Schematic of the cohort vaccination schedule for the first four doses of vaccines received. AdV = Adenovirus vector-based vaccine, mRNA = mRNA-based vaccine. Created in BioRender. Morse, R. (2025) https://BioRender.com/p69s364. **B** Nucleotide sequence-based cladogram of full-length spike proteins of SARS-CoV-2 Wu-1, SARS-CoV-2 variants and related sarbecoviruses. Tip labels are coloured according to the host species of each sarbecovirus: black = bat, blue = human, orange = pangolin. **C** Luciferase neutralisation assay of SARS-CoV-2 spike-pseudotyped lentiviruses, including SARS-CoV-2 Wu-1 (with D614G) after preincubation with serum samples taken 1 month post-fourth dose (*n* = 22), or with WHO reference sera (in a separate experiment, overlaid for comparison) in HeLa-ACE2 cells. 50% neutralising titres (NT50) of cohort sera against spike-pseudotyped lentiviruses are shown with individual points (blue = primary two-dose series with AZD1222, orange = primary two-dose series with BNT162b2, yellow square = Second WHO International Standard for anti-SARS-CoV-2 immunoglobulin (21/340, sera from individuals with Wu-1 infection only), green triangle = 1st International Standard 2022 Antibodies to SARS-CoV-2 variants of concern (21/338, sera from individuals vaccinated and infected with pre-Omicron VOC)) and bars indicating geometric mean titre (GMT) with 95% CI. Dotted lines indicate the minimum and maximum detection limits of the neutralisation assay. GMT is written below the minimum detection limit dotted line. *P*-values were calculated using the Friedman test and Dunn’s Multiple Comparisons test. Representative graph of *n* = 2 independent experiments, performed with two technical replicates each time. Neutralisation data for the WHO reference serum is from a separate experiment and is excluded in the calculation of GMT and statistical tests for the cohort serum. **D** Correlation of age and NT50 against spike-pseudotyped lentiviruses. Spearman r values and p-values were calculated using the nonparametric Spearman correlation test. *p < 0.05; **p < 0.01; ***p < 0.001; ****p < 0.0001; ns, *p* > 0.05.

To first investigate existing immunity in older individuals to Omicron VOCs compared to the original Wu-1 strain, we utilised an age- and sex-matched cohort (total n = 22, median age = 68.5 years, Table 1) of individuals in the UK who received a bivalent SARS-CoV-2 (Wu-1/BA.1) vaccine as their fourth dose in late 2022. These individuals had previously received a primary two-dose series of either AZD1222 (adenovirus vector-based) or BNT162b2 (mRNA-based) followed by a third mRNA-based monovalent booster dose 9-10 months after the first dose (Fig. 1A, Table 1, Supplementary Fig. 1). We performed a luciferase-based neutralisation assay in HeLa cells exogenously expressing human ACE2 using serum taken ~30 days (median: 31.5, IQR: 29–33) after the fourth bivalent vaccine dose, and assessed neutralisation capacity against lentiviral particles bearing the full-length spikes from SARS-CoV-2 Wu-1 (bearing the D614G substitution) and a range of Omicron VOCs (Fig. 1B, Supplementary Fig. 2). We assessed neutralisation against previously circulating variants (BA.2 and its descendants BA.4/BA.5, which share identical spike sequences) and descendants of BA.2 that had not yet circulated at the time of vaccination (BA.2.86 and XBB) (Supplementary Fig. 1). As expected, the highest levels of neutralisation were observed against SARS-CoV-2 Wu-1 (geometric mean titre (GMT) = 54410) (Fig. 1C, Supplementary Fig. 3A) despite the most recent vaccine including BA.1. Although significantly lower neutralisation of BA.2 and BA.4/5 was observed compared to SARS-CoV-2 Wu-1, neutralising titres remained high (GMT = 10084 and 7312, respectively). Four doses induced poor cross-neutralisation of BA.2.86 and XBB variants (GMT = 399 and 343, respectively), which circulated after administration of the fourth vaccine dose, consistent with reduced neutralisation of antigenically divergent spikes. Above age 60, there was no correlation between age and NT50 (the half-maximal inhibitory concentration) in this cohort (Fig. 1D). Only neutralisation against Wu-1 was significantly different between AZD1222 and BNT162b2 primary series groups (p < 0.05), although neutralising titres remained high for both (Supplementary Fig. 3B). There was a trend of older age in individuals who had received the BNT162b2 primary series (Supplementary Fig. 3C), which is most likely due to the prioritisation of older individuals for the first available vaccines in the UK (further information on UK vaccine administration in Materials and Methods: Cohort and Ethical Approval). We also observed a similar pattern of neutralisation of the same spikes by serum taken after 3 vaccine doses (Supplementary Fig. 4A).

**Table 1:** Cohort characteristics

Primary 2-dose series	Male, %	Female, %	Age, years (median, IQR)
**AZD1222 (AdV)**	8 (36.4%)	7 (32%)	67 (65, 70)
**BNT162b2 (mRNA)**	3 (13.6%)	4 (18%)	70 (70, 71)
**Total**	**11 (50%)**	**11 (50%)**	**68.5 (65.75, 70.25)**

Age- and sex-matched individuals (*n* = 22) vaccinated four times against SARS-CoV-2. All individuals received a primary two-dose series of either AZD1222 (adenovirus vector-based) or BNT162b2 (mRNA-based) against SARS-CoV-2 Wuhan-Hu-1 (Wu-1), followed by a 3rd dose (mRNA-based) against Wu-1 and a fourth bivalent dose (mRNA-based) against Wu-1 and B.1.1.529 (Omicron BA.1).

We also performed parallel neutralisation assays using two sets of WHO International Standard reference sera against the same panel of spike-pseudotyped lentiviruses (Fig. 1C). The first set of reference sera is the Second WHO International Standard for anti-SARS-CoV-2 immunoglobulin (called 21/340), which is sera pooled from seven individuals infected in the UK between May-August 2020 (pre-vaccine era, Wu-1 infection only). The second set of reference sera, the 1st International Standard 2022 Antibodies to SARS-CoV-2 Variants of Concern (called 21/338), is sera pooled from 265 individuals from the UK and Cameroon who were infected with SARS-CoV-2 Wu-1 or a pre-Omicron variant (including early 2020 variants, Alpha, Beta, or Delta), and who had also received one or more vaccine doses. Both WHO reference sera neutralised SARS-CoV-2 Wu-1 to a similar extent. However, the Wu-1 infection only WHO reference serum (21/340) was less effective at neutralising spikes from Omicron variants compared with the WHO reference serum from patients who had been naturally exposed to pre-Omicron VOCs and vaccinated (21/338).

### Diverse sarbecoviruses can use either human or R. sinicus ACE2 for cell entry

**Fig. 2:**
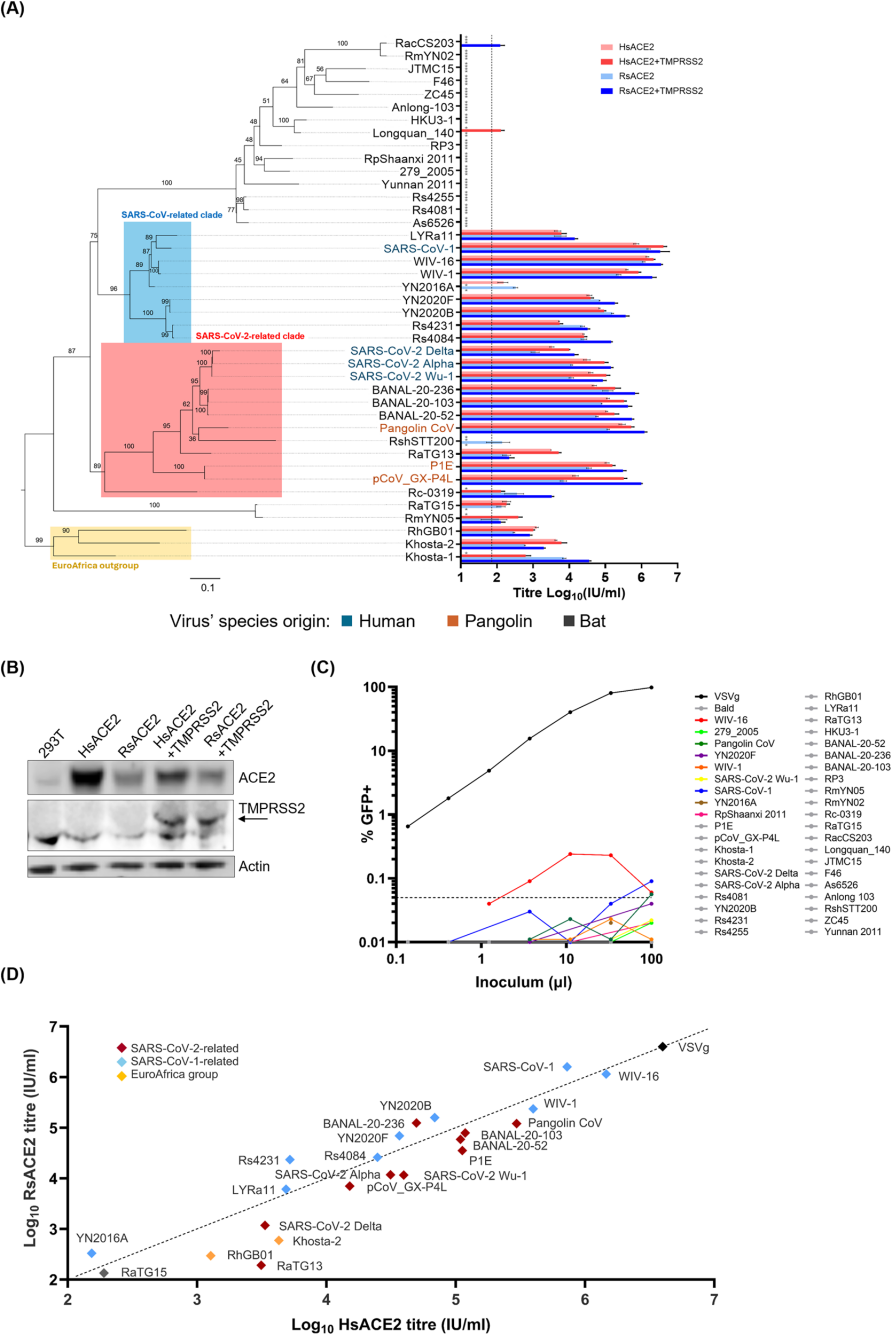
Entry of sarbecovirus spikes using human and *Rhinolophus sinicus* ACE2. **A** Nucleotide sequence-based phylogeny of the receptor-binding domains in sarbecovirus spikes isolated from bats (black text), pangolins (orange text) and humans (blue text). Ultrafast bootstrap support values are annotated on each internal branch. Different clades are highlighted on the cladogram: Red = SARS-CoV-2-like clade, blue = SARS-CoV-1-like clade, yellow = EuroAfrica outgroup. Mean viral titres of sarbecovirus spike-pseudotyped lentiviruses in HEK293T cells stably expressing ACE2 or ACE2 + TMPRSS2 from humans (light red or darker red) or Rhinolophus sinicus (light blue or darker blue bars). Dotted line represents minimum threshold for detection. Grey stars indicate that no entry was detected. Figure is representative of two independent biological repeats. **B** Expression of ACE2 and TMPRSS2 in untransduced HEK293T cells vs HEK293T cells transduced with human or *Rhinolphus sinicus* ACE2 or ACE2 + TMPRSS2 detected by western blot. Beta-actin = loading control. **C** Entry of sarbecovirus spike-pseudotyped lentiviruses into untransduced 293T cells (Positive control = VSVg). The dotted line represents the minimum threshold for detection. **D** Comparison of usage of human and *Rhinolophus sinicus* ACE2 by different sarbecovirus spikes. Blue = SARS-CoV-1-like clade, red = SARS-CoV-2-like clade, yellow = EuroAfrica Outgroup. The dotted line represents equal use of human and *Rhinolophus sinicus* ACE2.

We next wanted to investigate what level of cross-neutralisation vaccine-mediated immunity might offer against animal sarbecoviruses. To do this, we identified a panel of 40 different sarbecoviruses from bats, humans, and pangolins for which full-length genome sequences were available, and generated a phylogenetic tree to illustrate the evolutionary relationships between these viruses (Fig. 2A, Supplementary Table 1). Due to high levels of recombination between sarbecoviruses, different regions of the genome can have strikingly different phylogenetic relationships, so for this analysis, the tree was inferred using the nucleotide sequence corresponding to the RBD of each spike only.

The spike RBD sequences clustered into distinct clades, which have been previously described based on sequence and receptor binding^27,37,38^. The two main clades contained spikes that were either closely related to SARS-CoV-1 or those that were more closely related to SARS-CoV-2. Three spikes were members of the EuroAfrica clade, which contains viruses that have been isolated from both Europe and Africa, although the three spikes from our panel were all isolated from European bats^39,40^. There were also a number of other outliers that did not cluster with the above three clades; these spikes are known to contain deletions, which may affect their capacity to utilise ACE2 for cell entry^41,42^.

The ability for the spikes included in the phylogenetic analysis above to use either human or *Rhinolophus sinicus* ACE2 independently, or with the TMPRSS2 coreceptor, to enter human cells was then assessed using an entry assay with sarbecovirus spike-pseudotyped particles. Briefly, lentiviral particles encoding a GFP reporter^43^ were pseudotyped with the individual full-length sarbecovirus spikes, and entry of the pseudotyped particles into naïve 293 T cells, or 293 T cells exogenously expressing human or *Rhinolophus sinicus* ACE2 or ACE2 + TMPRSS2, was then quantified using flow cytometry to measure GFP-positive cells.

To confirm that any entry we observed in our assay was due to exogenous ACE2 expression, we first measured entry of the sarbecovirus spike-pseudotyped particles into naïve 293 T cells which express very low levels of endogenous ACE2 (Fig. 2B). In line with previous findings, the sarbecovirus spike-pseudotyped particles were generally unable to enter these cells (Fig. 2C), although spikes from SARS-CoV-1, WIV-16 and pangolin CoV facilitated a very low level of entry close to the detection threshold, likely reflecting use of a small amount of endogenous ACE2, or an ACE2-independent pathway. Nevertheless, since all three barely exceeded our approximate limit for detection (~0.05% GFP+ cells), we can conclude that most sarbecovirus spikes are generally unable to efficiently enter naïve 293 T cells.

We then examined the level of entry the sarbecovirus spike-pseudotyped particles displayed in 293 T cells exogenously expressing human or *Rhinolophus sinicus* ACE2 and found that many spikes from the SARS-CoV-1-like and SARS-CoV-2-like clades efficiently used human ACE2 for entry, often with a comparable efficiency to ACE2 from *Rhinolophus sinicus* (Fig. 2A, D). The coexpression of TMPRSS2 enhanced the entry of many of the spikes, particularly those from human CoVs (SARS-CoV-2 Wu-1, Alpha, and Delta, and SARS-CoV-1) (Fig. 2A). Interestingly, spikes from the SARS-CoV-1-like clade often had marginally higher entry titres in cells expressing *Rhinolophus sinicus* ACE2, whereas spikes from the SARS-CoV-2-like clade typically had higher entry titres in the cells expressing human ACE2 (Fig. 2D). Moreover, several bat and pangolin sarbecovirus spikes from the SARS-CoV-2-like clade facilitated higher levels of entry than any of the SARS-CoV-2 variants in our panel. While spikes from the EuroAfrica group exhibited some entry in the presence of exogenous ACE2, this was typically lower and more species-specific than spikes more closely related to SARS-CoV-1 or SARS-CoV-2. Other spikes that were more distantly related to SARS-CoV-1 and SARS-CoV-2 failed to use human or *Rhinolophus sinicus* ACE2 (Fig. 2A). These spikes may be able to use ACE2 orthologues from different *Rhinolophus* or other bat species, or they may use an unknown, ACE2-independent entry pathway^44,45^.

### Four COVID-19 vaccine doses in a population with mixed infection history are associated with high neutralisation of sarbecovirus spike-pseudotyped lentiviruses related to SARS-CoV-2

**Fig. 3:**
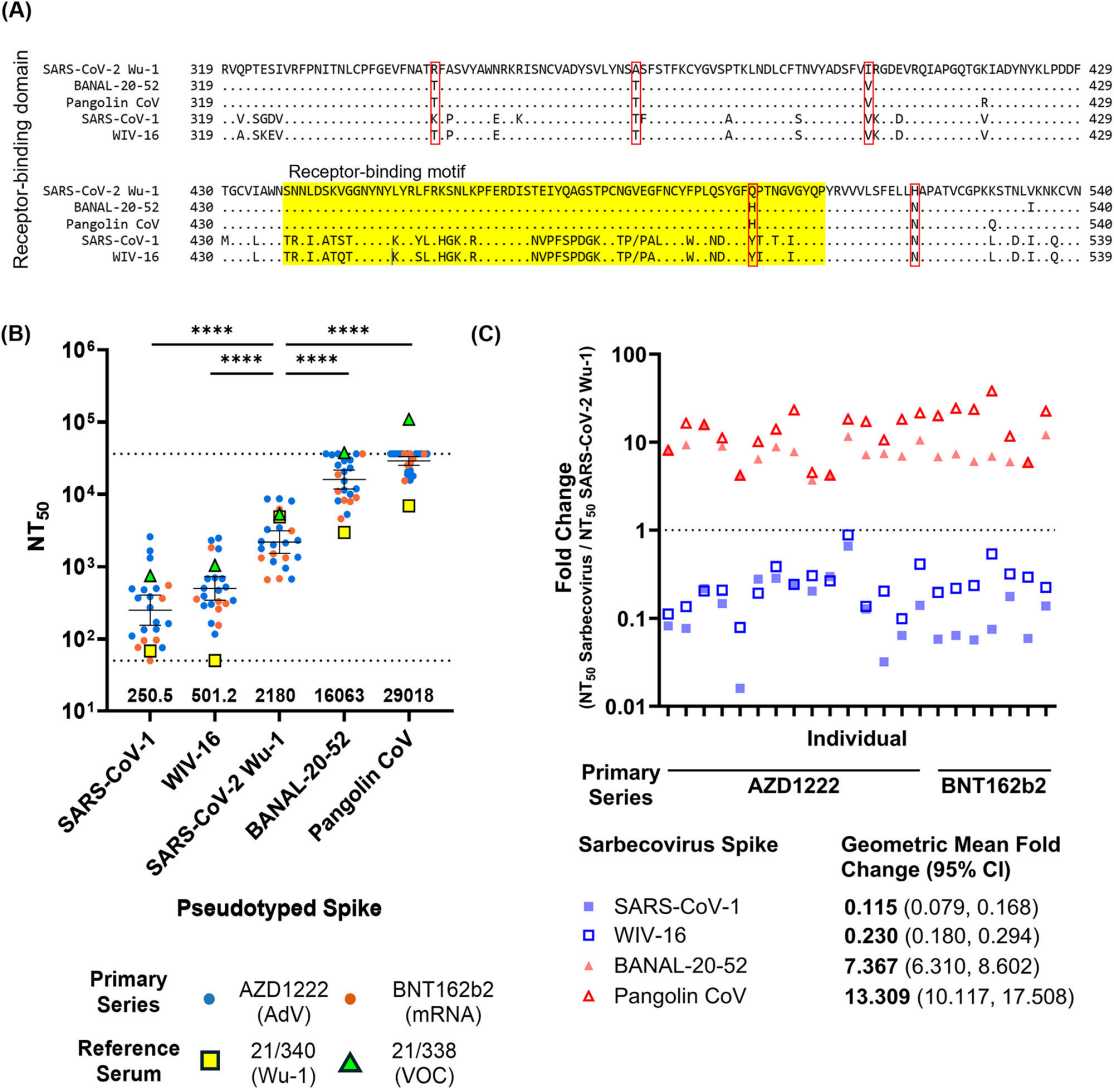
Neutralisation of sarbecovirus spikes by sera from individuals receiving four vaccinations. **A** Alignment of the amino acid sequence of the receptor-binding domains of the spikes used in this experiment. Yellow = Receptor-binding motif. Amino acid residues different between BANAL-20-52/pangolin CoV and SARS-CoV-2 Wu-1 are outlined in red. **B** GFP neutralisation assay of sarbecovirus spike-pseudotyped lentiviruses after preincubation with *n* = 22 serum samples taken 1 month post-fourth dose, or WHO reference sera (in a separate experiment), in HEK293T cells exogenously expressing human ACE2. 50% neutralising titres (NT50) of cohort sera against spike-pseudotyped lentiviruses are shown with individual points (blue = primary two-dose series with AZD1222, orange = primary two-dose series with BNT162b2, filled green square = Second WHO International Standard for anti-SARS-CoV-2 immunoglobulin (21/340), open green square = 1st International Standard 2022 Antibodies to SARS-CoV-2 variants of concern (21/338)) and bars indicating geometric mean titre (GMT) with 95% CI. Dotted lines indicate the minimum and maximum detection limits of the neutralisation assay for the experiment conducted using the *n* = 22 sera. GMT is written below the minimum detection limit dotted line. *P*-values were calculated using the Wilcoxon matched-pairs signed rank test relative to Wu-1. *p < 0.05; **p < 0.01; ***p < 0.001; ****p < 0.0001; ns, *p* > 0.05. Representative graph of *n* = 2 independent biological experiments, each performed with two technical replicates. Neutralisation data for the WHO reference serum is from a separate experiment and is excluded in the calculation of GMT and statistical tests for the cohort serum. **C** Fold change in neutralisation of lentiviruses pseudotyped with sarbecovirus spikes compared with SARS-CoV-2 Wu-1 (dotted line at y = 1) for each individual. Geometric mean fold change and 95% CI were calculated to compare neutralisation of each pseudotyped sarbecovirus spike to SARS-CoV-2 Wu-1.

Given that a number of the circulating bat and pangolin sarbecovirus spikes we tested appeared able to efficiently use human ACE2 for entry, and therefore could be candidates for potential spillover events, we next sought to investigate whether vaccines for SARS-CoV-2 could cross-neutralise a selection of these sarbecovirus spikes. BANAL-20-52 and pangolin CoV were selected for further investigation, as they are close relatives of SARS-CoV-2 and can efficiently use human ACE2 for entry (Figs. 1B, 2A). Specifically, the spike RBM of BANAL-20-52 and pangolin CoV are highly similar to the RBM of the SARS-CoV-2 Wu-1 spike, only varying at residue 498, while their RBDs differ by only six/seven amino acids, respectively (Fig. 3A). We also selected SARS-CoV-1 and a closely related bat sarbecovirus, WIV-16, because they both used human ACE2 effectively for entry (Fig. 2A), and we were interested in determining whether any potential vaccine-mediated cross neutralisation might extend to SARS-CoV-1-like sarbecovirus spikes. SARS-CoV-1 and WIV-16 accordingly have more divergent spike sequences, with 49% and 51% amino acid similarity in the RBM compared to SARS-CoV-2, respectively. Finally, for comparison, we used a Wu-1 spike lacking the D614G substitution to match the ancestral sequence.

To investigate cross-neutralisation potential, we incubated the aforementioned sera from quadruple-vaccinated individuals with approximately equivalent multiplicities of infection (MOIs) (~0.1) of the sarbecovirus spike-pseudotyped lentiviral particles before assessing entry into 293 T cells expressing exogenous human ACE2. We found that SARS-CoV-1 and WIV-16 were both poorly neutralised compared to SARS-CoV-2 Wu-1 (GMT = 250.5 and 501.2, respectively, compared to 2180 for Wu-1) (Fig. 3B, Supplementary Fig. 5A), consistent with their genetic distance from SARS-CoV-2. Yet rather surprisingly, BANAL-20-52 and pangolin CoV were neutralised significantly better than Wu-1, with NT50 values approximately 7.4-fold and 13.3-fold higher than that of Wu-1 (GMT = 16063 and 29018, respectively, compared to 2180) and with similar neutralisation breadth across all participants (Fig. 3B, C, Supplementary Fig. 5B, C). To further validate these unexpected results, we subsequently investigated whether this observation was also present in an additional, but very similar, small patient cohort (n = 10), and we again found that particles bearing the BANAL-20-52 and pangolin CoV spikes were significantly better neutralised than those bearing the spike from Wu-1 (Supplementary Fig. 6A–C). Similarly, we tested neutralisation of the same panel of spikes by serum taken after 3 vaccine doses from a group of patients from both cohorts and found a similar pattern of neutralisation (Supplementary Fig. 4B, C). To confirm that these differences in neutralisation could not be attributed to differences in spike expression between the sarbecovirus-pseudotyped particles, we performed western blots and found that total spike expression was roughly equivalent between all of the tested sarbecovirus-pseudotyped particles we tested (Supplementary Fig. 7).

Neutralisation of sarbecovirus spike-pseudotyped lentiviruses was also assessed using both sets of WHO reference sera mentioned previously (Fig. 3B). In line with our first assay, SARS-CoV-2 Wu-1 was neutralised similarly by both WHO reference sera. The Wu-1 infection only sera (21/340) showed minimal to no neutralisation of SARS-CoV-1 and WIV-16, and neutralisation of BANAL-20-52 and pangolin CoV was comparable to that of SARS-CoV-2 Wu-1. In contrast, the reference sera from patients who had been infected and vaccinated (21/338) displayed broader neutralisation of sarbecovirus spikes, including increased neutralisation of BANAL-20-52 and pangolin CoV relative to SARS-CoV-2 Wu-1, resembling the pattern observed in sera from our cohort of individuals receiving third and fourth vaccine doses. Finally, we did not observe any impact of primary series or age on neutralisation of each sarbecovirus spike (Supplementary Fig. 4B, C).

### Assessment of prior infection and spike-binding responses across vaccination time points

We then sought to determine whether prior infection had an effect on neutralisation by all available remaining sera in both cohorts across time points. We tested total IgG binding collected one month post-dose 2 (*n* = 8), post-dose 3 (*n* = 16), and post-dose 4 (*n* = 22) (Supplementary Fig. 8A–C). Binding responses were assessed against SARS-CoV-2 nucleocapsid (N) and the RBD of SARS-CoV-2 Wu-1 and Omicron BA.1 spikes. Seropositivity thresholds were defined as mean fluorescence intensity values three standard deviations above the mean (1473.8 for N, 411.91 for Wu-1 RBD, and 729.1 for BA.1 RBD). Prior infection was defined as the presence of anti-N IgG above the seropositivity threshold. Using this criterion, a minority of individuals were seropositive prior to their fourth dose (2/8 post-dose 2; 2/16 post-dose 3), whereas most individuals were anti-N–positive post-dose 4 (16/22) (Supplementary Fig. 8A).

Binding responses to SARS-CoV-2 Wu-1 and BA.1 RBD increased with each additional vaccine dose (Supplementary Fig. 8B, C). Comparable binding to BA.1 RBD was observed in individuals with serological evidence of prior infection and those without detectable anti-N antibodies following bivalent vaccination, and there was no correlation between anti-N binding and increased neutralisation for any of the spikes tested across both panels (Supplementary Fig. 8D, E). Neutralisation assays performed using post-dose 3 sera (n = 16) against the same panel of VOC and sarbecovirus spike-pseudotyped lentiviruses revealed neutralisation potency and breadth comparable to that observed in post-dose 4 sera (Supplementary Fig. 4). These findings suggest that vaccination, and not infection, was responsible for increased breadth of cross-neutralisation against sarbecoviruses and are discussed below in more detail.

## Discussion

In this study, we investigated whether neutralising antibody responses elicited by four doses of COVID-19 vaccines (three against ancestral Wu-1 spike and the fourth against Omicron lineage spikes) in older adults could offer cross-neutralisation against spikes from SARS-CoV-2 VOCs and spikes from other bat and pangolin sarbecoviruses that are able to readily use ACE2 to enter human cells in vitro. Compared to the original SARS-CoV-2 Wu-1 spike-pseudotyped particles, we observed reduced cross-neutralisation of Omicron VOC spikes, consistent with the evolution of their spike proteins in response to population-level selective pressure from vaccines and prior infection.

As expected, bivalent vaccination induced significant neutralisation of BA.2 and BA.4/5, which was expected given that BA.2 and BA.1 differ by only six amino acids in their RBD. However, despite bivalent vaccination, the highest neutralisation titres were observed against Wu-1, consistent with immune imprinting following repeated exposure to the ancestral spike. In contrast, our tested sera exhibited poor neutralisation of BA.2.86 and XBB VOCs, consistent with their antigenic distance from Wu-1 and emergence after the administration of the fourth vaccine dose.

Beyond SARS-CoV-2 VOCs, we observed markedly reduced neutralisation of spikes from SARS-CoV-1 and the closely related bat sarbecovirus WIV-16, consistent with their phylogenetic distance from SARS-CoV-2 (Fig. 3A). We were therefore surprised to observe that individuals receiving a bivalent (Wu-1/BA.1) fourth dose generated neutralising titres over 7-fold higher against BANAL-20-52 and pangolin CoV as compared to SARS-CoV-2 Wu-1. This finding was unexpected, given the close genetic similarity between these spikes and represents a central and novel observation of this study.

One reason this finding was particularly unexpected is that spikes from BANAL-20-52 and pangolin CoV exhibit only one amino acid difference from the spike from SARS-CoV-2 Wu-1 in the RBM (at position 498). Although substitutions in the RBM are known to be significant determinants of ACE2 binding affinity and immune evasion^11,12^, the magnitude of change in neutralisation between these CoVs and SARS-CoV-2 Wu-1 was unanticipated. Whereas increased neutralisation of RaTG13 has been observed relative to SARS-CoV-2, likely because of its decreased affinity for human ACE2^46,47^, the RBDs of pangolin CoV and BANAL-20-52 have both been observed to bind the human ACE2 with similar, or higher, efficiency^28,48^. This suggests that differences in neutralisation may be due to other changes in the RBD, or even in other parts of the spike.

Indeed, it is plausible that structural or conformational differences such as altered RBD exposure or spike trimer configuration, potentially modulated by allosteric interactions across the spike (from N-terminal domain to S2 subunit), could play a critical role in the pattern of neutralisation profiles that we observe in our current study^49,50^. Future studies employing domain-swapping of sarbecovirus spikes, or structural studies such as cryo-electron microscopy, could reveal more about which regions or conformations of sarbecovirus spikes influence neutralisation.

Our findings suggest that breadth of sarbecovirus neutralisation is driven primarily by vaccine-induced immune maturation rather than specifically by Omicron infection. Neutralisation breadth against BANAL-20-52 and pangolin CoV was already evident in sera collected after the third vaccine dose, where the first three vaccine doses targeted only the spike of SARS-CoV-2 Wu-1 (Supplementary Fig. 4). The fold-difference in 50% neutralising titre of the sarbecovirus spikes compared to Wu-1 was comparable to that observed following the fourth bivalent dose, despite a higher frequency of anti-nucleocapsid seropositivity after dose four (Supplementary Fig. 8A). This indicates that exposure to Omicron through infection or bivalent vaccination is not required to achieve enhanced cross-sarbecovirus neutralisation. Instead, these findings are consistent with recall and maturation of antibody responses primed by Wu-1-based vaccination. In support, the WHO reference sera collected from patients who had been vaccinated and infected with a non-Omicron variant (21/338) showed a similar pattern of increased neutralisation of BANAL-20-52 and pangolin CoV, whereas the reference sera from those who had only been infected did not (21/340). This interpretation aligns with previous studies demonstrating that repeated vaccination drives increased somatic hypermutation, greater tolerance to RBD substitutions, and expansion of broadly reactive B cell populations, particularly in older adults^51,52^. Together, these data support a model in which repeated exposure to the SARS-CoV-2 spike refines immune memory towards broader sarbecovirus recognition, rather than inducing solely variant-specific responses.

Although prior exposure to human seasonal coronaviruses such as OC43, 229E, and HKU1 can elicit cross-reactive antibodies and T-cell responses to conserved SARS-CoV-2 epitopes, these responses generally show limited neutralising activity against SARS-CoV-2^53,54^. Such pre-existing immunity may nonetheless shape subsequent vaccine responses by priming recall of conserved targets^55^. However, seasonal coronaviruses are phylogenetically distant from sarbecoviruses, and cross-reactive responses induced by endemic CoVs are unlikely to confer meaningful neutralisation of non-human sarbecoviruses. Consistent with this, our data support a model where vaccine-elicited, RBD-directed immunity - rather than pre-existing immunity to endemic seasonal CoVs - is the primary driver of the breadth of neutralisation observed in this study.

The neutralisation patterns observed in our cohorts were further supported by analysis of the WHO reference sera. Sera from individuals infected early in the pandemic with SARS-CoV-2 Wu-1 (21/340) exhibited limited sarbecovirus neutralisation breadth, whereas sera pooled from individuals with combined infection and vaccination histories (21/338) demonstrated broader neutralisation, including enhanced activity against BANAL-20-52 and pangolin CoV. The similarity between the WHO reference sera and sera from our cohort of third- and fourth-dose vaccine recipients reinforces the conclusion that repeated or heterologous antigenic exposure increases breadth of cross-neutralising antibody responses. Notably, anti-nucleocapsid seropositivity did not correlate with neutralisation titres, further supporting the role of vaccine-elicited, RBD-directed immunity in driving cross-sarbecovirus neutralisation.

Our findings differ from some previous reports describing limited or equivalent neutralisation of pangolin sarbecoviruses relative to SARS-CoV-2^30,36,56^. Many factors, including differences in cohort age, infection history, and vaccine dose number, could explain these discrepancies. Importantly, many prior studies assessed sera after one or two vaccine doses, whereas our analyses were performed following three or four doses, a factor which we expect to enhance neutralisation breadth^51,52^. Methodological differences may also contribute; while some studies measured neutralisation at a fixed serum dilution, we calculated NT50 values across a broad dilution range, allowing more sensitive discrimination of neutralisation potency, particularly in samples exhibiting high neutralisation activity^57^.

This study does have several limitations. Our focus on an older adult cohort may limit generalisability to younger populations; this cohort is representative of a clinically relevant and high-risk demographic. Additionally, the consistency in breadth of cross-neutralisation across all participants despite differing primary series (Fig. 3C), as well as the inclusion of WHO reference sera and a second small cohort with similar patterns of neutralisation post-dose 3 and 4, mitigates some concerns about NT50 variability and the effects of infection on cross-neutralisation. The number of sarbecovirus spikes tested was constrained by serum availability, and broader panels incorporating additional viruses with diverse ACE2 binding properties would further refine understanding of cross-neutralisation. Finally, we chose to use a pseudotyped lentivirus-based assay instead of using replication-competent, full-length viruses - previous work across a broad range of pseudotyped virus systems has shown that they are a valid alternative and typically offer very similar neutralisation results^58,59^.

In summary, our findings highlight the capacity of repeated COVID-19 vaccination to drive unexpectedly broad neutralisation of certain animal sarbecoviruses, even in the absence of variant-matched exposure. The enhanced neutralisation of BANAL-20-52 and pangolin CoV observed here suggests that existing vaccines, particularly with repeated boosters, may provide a foundation for broader protection against other animal sarbecoviruses. Further research should investigate the basis for increased neutralisation sensitivity of BANAL-20-52 and pangolin CoV spikes that we observed, including exploring the role of other spike domains or certain spike conformations, as this may be key to identifying the determinants of sarbecovirus sensitivity to neutralisation and unlocking improvements to future vaccines. While specific to an older cohort, our data support the value of booster vaccination in enhancing cross-sarbecovirus immunity and underscore its potential role in pandemic preparedness and the protection of vulnerable populations. Moreover, this has important implications for the design of next-generation sarbecovirus vaccines - including the incorporation of conserved spike epitopes into multivalent or mosaic formulations spanning existing and emerging lineages that could provide broader protection against adverse outcomes from both circulating VOCs and zoonotic sarbecoviruses with pandemic potential.

## Methods

### Cohort and ethical approval

We selected 22 serum samples from participants who had received four vaccine doses. The cohort median age was 68.5 years (Table 1), and selected participants were sex- and age-matched. For the primary two-dose series, seven individuals received BNT162b2 mRNA-based vaccines and 15 received AZD1222 adenovirus vector-based vaccines. Participants were recruited through the NBR118 Study at the Cambridge NIHR BioResource Centre. Individuals consented to providing post-vaccination biological samples as well as relevant clinical data. The study was approved by the East of England – Cambridge Central Research Ethics Committee (17/EE/0025) on April 28th, 2020.

In the UK, the vaccination response began with the licensing of the mRNA-based Pfizer vaccine (BNT162b2), initially offered to residents of care homes and individuals over 80 years old. Subsequently, the adenovirus vector-based Oxford-AstraZeneca ChAdOx1 nCoV-19 vaccine (AZD1222) was offered to the next priority group. Despite initial success, neutralising antibody responses were observed to wane over time, leading to the recommendation of booster doses. In the UK, official guidelines recommended an interval of up to 12 weeks between the first and second doses based on efficacy estimates and in order to protect as many people as possible. Many individuals aged over 70 have now received at least four doses, with ongoing vaccinations enhancing both the potency and breadth of the neutralising antibody response against SARS-CoV-2^51^. Additionally, two sets of WHO reference sera (sourced from NIBSC) were used for comparison and to calibrate our neutralisation assays (Fig. 1C, 3B). The first set of reference sera is the Second WHO International Standard for anti-SARS-CoV-2 immunoglobulin (called 21/340), which is sera pooled from seven individuals infected in the UK between May-August 2020 (pre-vaccine era, Wu-1 infection only). The second set of reference sera, the 1st International Standard 2022 Antibodies to SARS-CoV-2 Variants of Concern (called 21/338), is sera pooled from 265 individuals from the UK and Cameroon who were infected with SARS-CoV-2 Wu-1 or a pre-Omicron variant (including early 2020 variants, Alpha, Beta, or Delta), and who had also received one or more vaccine doses.

### Phylogeny and alignment

We identified a number of sarbecoviruses for which the full genome sequence was available (Supplementary Table 1). For these sarbecoviruses, the nucleotide sequence was aligned using MAFFT (localpair option)^60^, and trees were inferred using IQ-TREE2 under a GTR + F + R10 substitution model with 10000 ultrafast bootstrap iterations to determine node support^61^. Phylogenetic trees were visualised using FigTree (https://github.com/rambaut/figtree/). Amino acid sequences of sarbecovirus spike proteins were aligned using MAFFT v7.526, implemented via Python 3.8.8 with Biopython 1.83 for sequence processing^62^. The custom Python script is available at https://github.com/baseten418/shirley.

### Cell maintenance

HEK293T cells, a human kidney cell line, were used in experiments and for the generation of cell lines and were obtained from existing stocks from the Rihn laboratory. All HEK293 T-derived cell lines were cultured in Dulbecco’s Modified Eagle Medium (DMEM) supplemented with 10% foetal bovine serum (FBS) and 50 mg/ml gentamicin and were grown at 37 °C in 5% CO_2_. Cells were routinely screened for mycoplasma. HeLa-ACE2 cells (a gift from J. Voss) were maintained in DMEM supplemented with 10% FBS and 1% penicillin-streptomycin and grown at 37 °C in 5% CO2.

### Generation of ACE2- and ACE2+TMPRSS2-expressing cell lines

HEK293T cell lines exogenously expressing human (HsACE2) (NP_001358344.1) or *Rhinolophus sinicus* (RsACE2) (XP_019601896.1) ACE2 were generated via lentiviral transduction using the pLV-EF1a-IRES-hygro expression vector (Addgene #85134). To generate ACE2 + TMPRSS2 cell lines, the cell lines stably expressing ACE2 were subsequently transduced with human TMPRSS2 (NP_001128571.1, in pLV-EF1a-IRES-blasti (Addgene #85133)) or *Rhinolophus sinicus* TMPRSS2 (XP_019601335.1, in pLV-EF1a-IRES-puro expression vector (Addgene #85132)). Plasmid sequences were verified by Plasmidsaurus using Oxford Nanopore Technology with custom analysis and annotation.

Lentiviruses were produced by co-transfecting HEK293T cells with 5 μg of the pLV-EF1a-IRES-hygro ACE2 expression vector, 5 μg of a lentiviral transfer and packaging plasmid (NL4.3 GagPol), and 1 μg of VSVg (glycoprotein from vesicular stomatitis virus) using polyethylenimine (PEI) in serum-free DMEM. Cells were seeded at 1/3 confluence 24 h prior to transfection. Viral supernatant was collected at 48 h post-transfection and filtered (0.45 µm), and the filtered supernatant was then used to transduce HEK293T cells in 24-well plates by spinoculation (1 h, 1600 RPM, room temperature), after which cells were incubated at 37 °C in 5% CO_2_. The transduced cells were subsequently selected and maintained in 200 µg/ml hygromycin for at least seven days before use, and mock-transduced controls confirmed successful selection.

Expression of ACE2 in the 293T-HsACE2 and HeLa-ACE2 cell lines was confirmed using flow cytometry (Supplementary Fig. 9).

### Western blot analyses

To determine expression of ACE2 and TMPRSS2 in our cell lines, cell pellets were resuspended in protein sample buffer (12.5% glycerol, 175 mM Tris-HCl [pH 8.5], 2.5% SDS, 70 mM 2-mercaptoethanol, 0.5% bromophenol blue). Proteins were separated on 4% to 12% Bis-Tris polyacrylamide gels and transferred onto nitrocellulose membranes. Blots were probed with either anti-ACE2 (21115-1-AP; Proteintech), anti-TMPRSS2 (sc-515727; Santa Cruz Biotechnology), or anti-β-actin as a loading control (66009-1-1 g; Proteintech), then were probed with fluorescently labelled goat anti-mouse (SA5-10176; Invitrogen) or goat anti-rabbit (SA5-10036; Invitrogen) secondary antibodies and scanned using a LiCor Odyssey scanner.

To compare spike incorporation, supernatants containing sarbecovirus spike-pseudotyped lentiviral particles were spun at 13,000xg for two hours through 20% sucrose in PBS, and the resulting pellet was resuspended in 50 μL protein sample buffer (12.5% glycerol, 175 mM Tris-HCl [pH 8.5], 2.5% SDS, 70 mM 2-mercaptoethanol, 0.5% bromophenol blue). 10 μl of each sample was separated on 4% to 12% Bis-Tris polyacrylamide gels and transferred onto nitrocellulose membranes. Blots were probed with either anti-SARS-CoV-2 S2 (PA1-41165; Invitrogen) or anti-p24 (183-H12-5C hybridoma; NIH AIDS reagents program), then were probed with fluorescently labelled goat anti-mouse (SA5-10176; Invitrogen) or goat anti-rabbit (SA5-10036; Invitrogen) secondary antibodies and scanned using a LiCor Odyssey scanner.

Uncropped Western blots are presented.

### Generation of luciferase-expressing VOC spike-pseudotyped lentiviruses

Pseudotyped particles representing the original SARS-CoV-2 strain (Wu-1) (MN908947.3) and Omicron lineage VOCs were generated as previously described^63^. In short, specific amino acid substitutions were introduced into the pcDNA3.1 SARS-CoV-2 S D614G plasmids. The pseudotyped particles were created using a triple plasmid transfection system, where the Spike-expressing plasmid, along with a lentiviral packaging vector (p8.91), and a luciferase expression vector (psCSFLW), were transfected into HEK293T cells using the Fugene HD transfection reagent (Promega). After 48 h, the viruses were collected and stored at −80 °C. Individual TCID50s were determined by titrating the viruses on HeLa-ACE2 cells.

### Neutralisation assay of luciferase-expressing VOC spike-pseudotyped lentiviruses

Neutralisation assays were conducted using HeLa cells expressing exogenous human ACE2 and SARS-CoV-2 spike-pseudotyped lentiviral particles carrying the luciferase gene. Due to high neutralisation values post-dose 4 for SARS-CoV-2 Wu-1 D614G and BA.2, heat-inactivated human serum samples at a starting dilution of 1:80 were titrated using an eight-point, three-fold dilution series in DMEM. For SARS-CoV-2 BA.4/5, BA.2.86, and XBB, and for all spike-pseudotyped lentiviruses tested against post-dose 3 sera, heat-inactivated human serum samples at a starting dilution of 1:40 were titrated using an eight-point, three-fold dilution series in DMEM. Pseudotyped particles were then incubated with sera in duplicate for 1 h at 37 °C. Pseudotyped particles were diluted based on the calculated TCID50 to produce between 300,000–400,000 RLU in the BrightGlo Luciferase Assay. Controls were included on each plate, allowing normalisation to 100% inhibition of virus (cells only, no pseudotyped virus or serum) and 0% inhibition of virus (cells and pseudotyped virus, no serum). After the incubation, HeLa-ACE2 cells were added to each well. After 48 h at 37 °C and 5% CO_2_, luminescence was measured using the BrightGlo Luciferase Assay System (Promega, UK), and neutralisation was calculated relative to the controls. After normalisation to cells only (background) and virus only, the cutoff value was the lowest dilution factor of 40 (BA.4/5, BA.2.86, and XBB) or 80 (Wu-1 D614G and BA.2) for post-dose 4 sera, and 40 for post-dose 3 sera. The 50% neutralisation titre (NT50) was determined using the half-maximal inhibitory concentration values of individual serum samples from their serial dilutions, normalised to control infections, and reported as geometric mean titres. Statistical comparisons among groups were performed using the Friedman test and Dunn’s Multiple Comparisons test. All statistical analyses were conducted on Prism version 10.4.1. Experiments were carried out with two technical replicates and were repeated to give two biological replicates.

### Generation of sarbecovirus spike-pseudotyped lentiviruses

Lentiviruses pseudotyped with sarbecovirus spikes were produced by co-transfection of 5 μg of CSGW reporter lentiviral vector that encodes GFP^43^, 5 μg of NL4.3 GagPol, and 8 μg of the full-length sarbecovirus spike (codon optimised, in expression vector pcDNA3.1) into HEK293T cells. After 48 h, the sarbecovirus spike-pseudotyped particles were harvested and passed through 0.45 μm filters. Accession IDs for each sarbecovirus spike protein investigated are listed in Supplementary Table 1.

### Entry/neutralisation assays of sarbecovirus spike-pseudotyped lentiviruses

24 h before transduction, 293T-HsACE2 (Hs, *Homo sapiens*) or 293T-RsACE2 (Rs, *Rhinolophus sinicus*) cells were seeded into 96-well plates at 15,000 cells per well.

For entry assays, sarbecovirus spike-pseudotyped particles were titrated onto cells using 7-point, three-fold serial dilutions in DMEM. Transduced cells were spinoculated for 1 h at 1600 RPM and 20 °C, then incubated for 48 h at 37 °C in 5% CO_2_. Infected cells were fixed in 4% PFA and stored at 2–5 °C for subsequent flow cytometry analysis. The entry assay data presented here are representative of two biological replicates.

For neutralisation assays, heat-inactivated human serum samples at a starting dilution of 1:50 were titrated in duplicate using seven-point, three-fold serial dilutions in DMEM, then mixed with sarbecovirus spike-pseudotyped particles (which were titrated beforehand to determine a fixed concentration that would give an MOI of ~0.1), followed by incubation at 37 °C and 5% CO_2_ for 1 h. Controls were included on each plate, allowing normalisation to 100% inhibition of virus (cells only, no pseudotyped virus or serum) and 0% inhibition of virus (cells and pseudotyped virus, no serum). Then, the incubated serum/pseudotyped particle mixtures were added to the pre-seeded 293T-HsACE2 cells and spinoculated for 1 h at 1600 RPM and 20 °C, then incubated for 48 h at 37 °C in 5% CO_2_. Infected cells were fixed in 4% PFA and stored at 2-5 °C for flow cytometry analysis. Experiments were conducted in duplicate and were repeated to give two biological replicates. For the neutralisation assays comparing Wu-1 to VOCs, we chose to use Wu-1 with D614G because we were interested in comparing widely circulating strains. However, when performing the assays with different sarbecovirus spikes, we chose to use the Wu-1 without D614G because this is the sequence included in the vaccines and is more closely related to the bat and pangolin spikes. We analysed the neutralisation of the two Wu-1 spikes used in the luciferase-based and GFP-based assays and found that there was a strong degree of correlation (Supplementary Fig. 3D).

### Flow cytometry and other analyses

Flow cytometry was conducted using a Cytek® Guava® easyCyte™ Flow Cytometer to measure GFP signal. Neutralisation was calculated relative to cells-only and virus-only (without serum) controls. NT50 values for each group were reported as geometric mean titres, and statistical comparisons between SARS-CoV-2 Wu-1 and the other groups were performed using the Wilcoxon matched-pairs signed rank test. All statistical analyses were conducted on Prism version 10.4.1.

### SARS-CoV-2 variant prevalence dynamics

Variants of circulating SARS-CoV-2 were queried from NCBI Genbank via LAPIS API and GISAID using the outbreak.info R API. Subclades of WHO-assigned Variants of Concern (VOCs) were assigned their WHO name and Nextstrain-assigned clade.

### Flow cytometry to quantify ACE2 and TMPRSS2 expression of different cell lines

HEK293T-ACE2 (HsACE2), and HeLa-ACE2 (HeLa-ACE2) cells were maintained in their respective cell culture media at 37 °C with 5% CO2 in a 6-well plate (Corning). Cells were dissociated using trypsin for 5 min before the addition of the respective cell culture media to resuspend the cells. Cells were spun down before they were transferred into round-bottom 96-well plates and incubated with Live/Dead Fix Near IR (ThermoFisher Scientific; #L34994; 1:1000 dilution) in PBS for 15 min at room temperature. Cells were then resuspended in 100 µl of 4% paraformaldehyde (PFA) after two washes with PBS and incubated for 20 min at room temperature in the dark. After two washes in FACS buffer (PBS + 2.5% FBS + 2 mM EDTA), cells were stained with 100 µl of Anti-Human TMPRSS2 Alexa Fluor 488-conjugated antibody (R&D Systems; #FAB10723G; 1:500 dilution) and Anti-Human ACE2 primary antibody (R&D Systems; #AF933; 1:500 dilution) overnight at 4 °C. After two washes in FACS buffer, cells were resuspended in 100 µl of Donkey Anti-Goat IgG H&L secondary antibody (Abcam; #ab150131; 1:1000 dilution) in FACS buffer for 1 h at 4 °C in the dark. After two further washes in FACS buffer, the stained samples were then acquired on a LSRFortessa™ X-20 (BD Biosciences), and analysis was performed on FlowJo (BD Biosciences).

### Binding assay for infection history

We used a previously described Luminex-based flow cytometry assay to measure binding IgG antibodies against SARS-CoV-2 nucleocapsid (N), Wu-1 D614G and Omicron BA.1 receptor-binding domains^64–66^. We validated the assay using pre-pandemic serum samples and WHO reference sera, which permitted the determination of cut-off values for each binding measurement, defined as the mean + 3 standard deviations. We defined positive total IgG against SARS-CoV-2 Wu-1 nucleocapsid, Wu-1 D614G RBD, and BA.1 RBD, as a mean fluorescence intensity above the cut-offs of 1473.8, 411.91, and 729.1, respectively. Previous SARS-CoV-2 infection was defined as positive anti-N IgG above the cut-off of 1473.8 MFI.

## Supplementary information

Supplementary Information

## Data Availability

Data is provided within the manuscript and supplementary information files. Uncropped western blots are presented as supplementary Figs. 10–12.
