# Supplementary material for: COVID-19 vaccination induces cross-neutralisation of sarbecoviruses related to SARS-CoV-2
Source: NPJ Vaccines. 2026 Jul 1;11:125. doi: 10.1038/s41541-026-01469-x (PMC13324355; doi:10.1038/s41541-026-01469-x)
Supplement: Supplementary file 1 — Supplementary Information [file 41541_2026_1469_MOESM1_ESM.pdf]

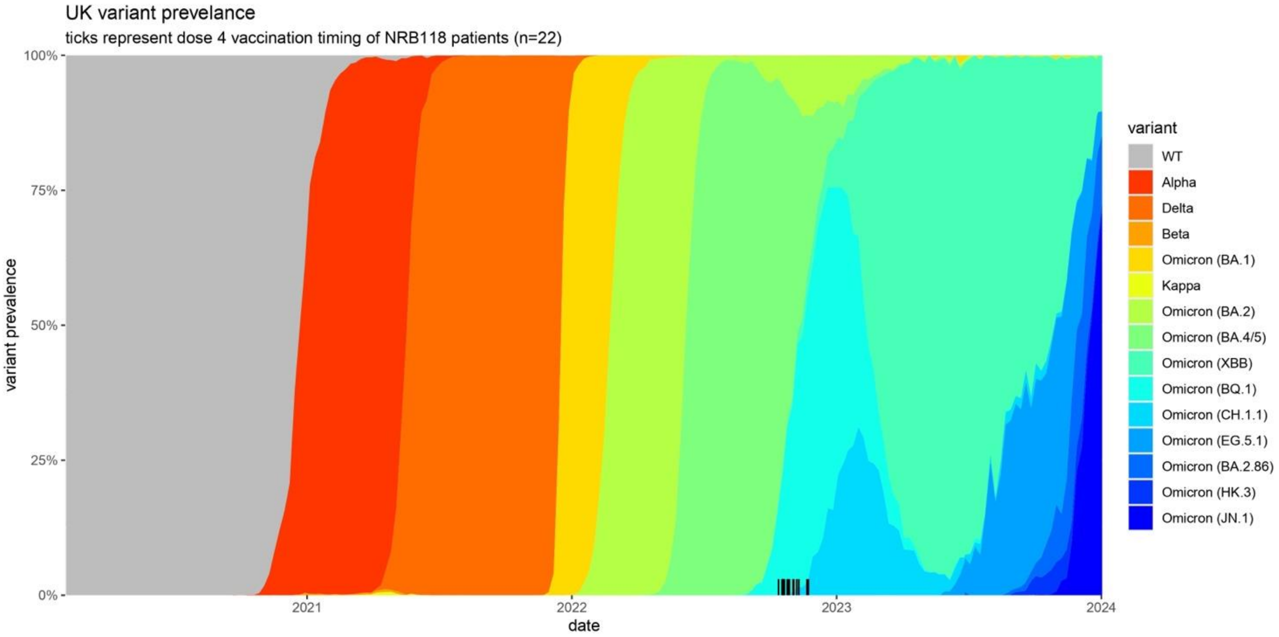

Supplementary Figure 1: SARS-CoV-2 variant prevalence dynamics. Circulation of SARS-CoV-2 variants in the UK from pandemic start through to December 2023. Black lines indicate the sampling dates of the samples taken after fourth dose used in the cohort of n = 22 individuals.

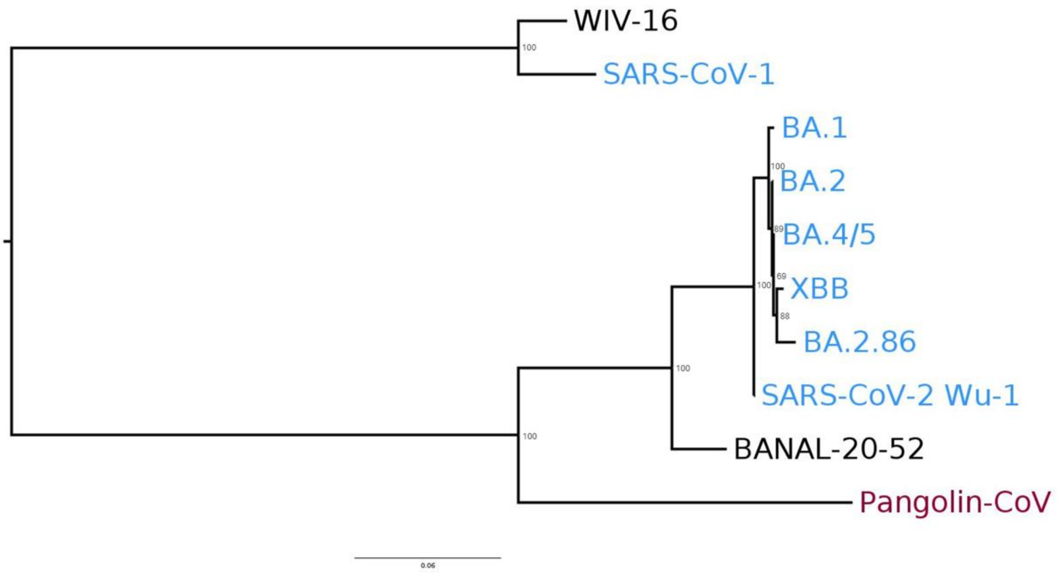

Supplementary Figure 2: Nucleotide sequence-based phylogenetic tree of full-length spikes of SARS-CoV-2 Wu-1, SARS-CoV-2 variants and related sarbecoviruses. Branch lengths drawn to scale. Bootstrap support for each node is indicated. Tip labels are coloured according to the host species of each sarbecovirus: black = bat, blue = human, red = pangolin.

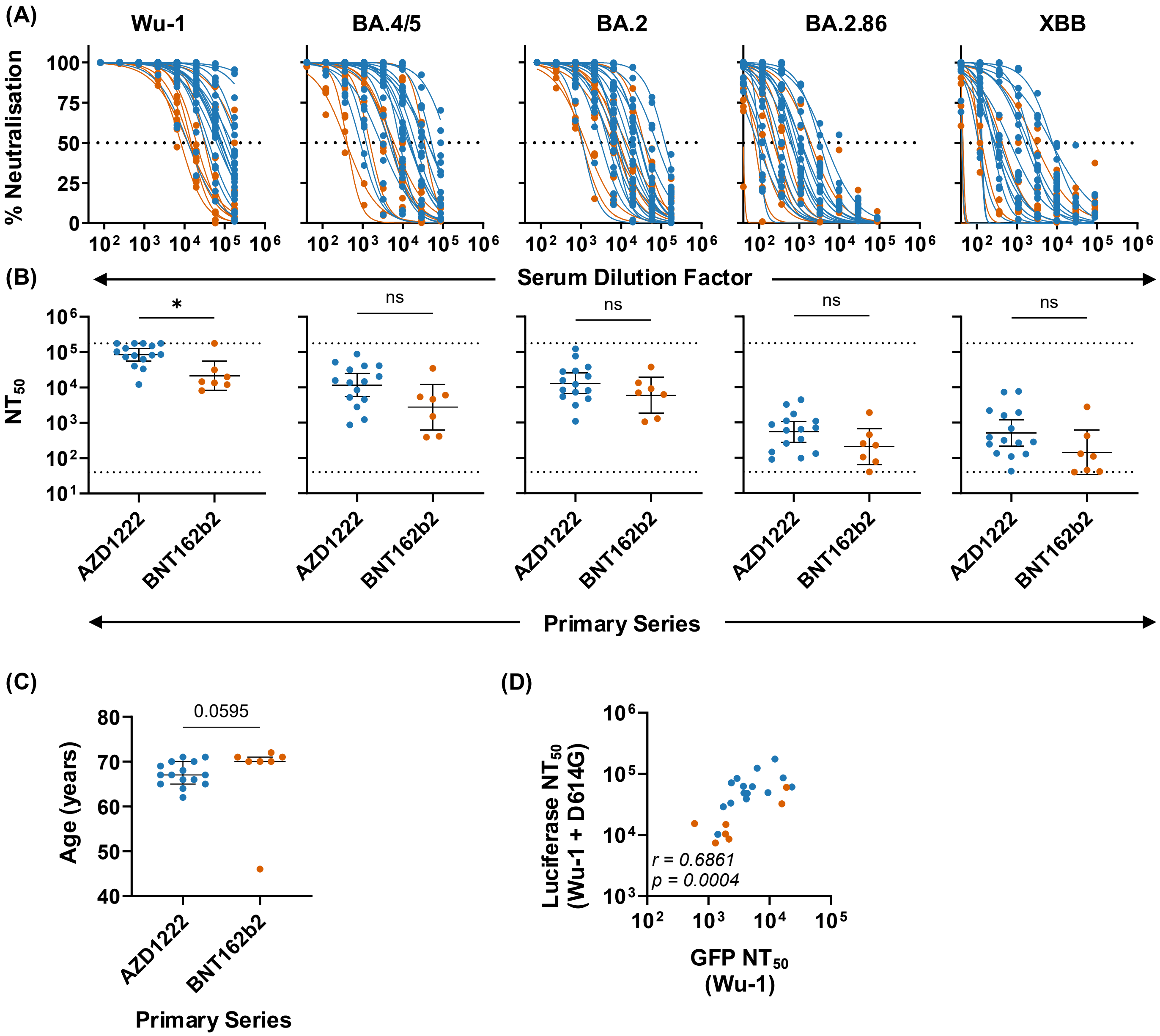

Supplementary Figure 3: Neutralisation curves and comparisons among age, primary series and correlation with GFP-based readout. (A) Neutralisation of SARS-CoV-2 spike-pseudotyped luciferase lentiviruses (Wu-1 + D614G, BA.4/5, BA.2, BA.2.86, XBB) after preincubation with n = 22 serum samples taken 1 month post-fourth dose in HeLa cells stably expressing human ACE2. Values were normalised to cells-only and virus-only wells. Blue points indicate a primary two-dose series with AZD1222 and orange points indicate a primary two-dose series with BNT162b2. The dotted line at y = 50 indicates the point at which 50% of the pseudotyped virus was neutralised by serum sample. (B) 50% neutralising titre (NT50) stratified by primary vaccine series (AZD1222 or BNT162b2) with bars indicating geometric mean titre (GMT) with 95% CI. P-values were calculated using a Mann Whitney test. (C) Ages of individuals in the cohort stratified by primary vaccine series. Bars indicate median age with interquartile range. (D) Correlation of NT50 generated by GFP (x-axis) and luciferase (y-axis) neutralisation assays. For luciferase assays, SARS-CoV-2 Wu-1 included the D614G mutation and were performed on HeLa-ACE2 cells, whereas GFP assays did not include the D614G mutation and were performed on HEK293T-ACE2 cells. Spearman r values and p-values were calculated using the nonparametric Spearman correlation test. *p < 0.05; **p < 0.01; ***p < 0.001; ****p < 0.0001; ns, p > 0.05.

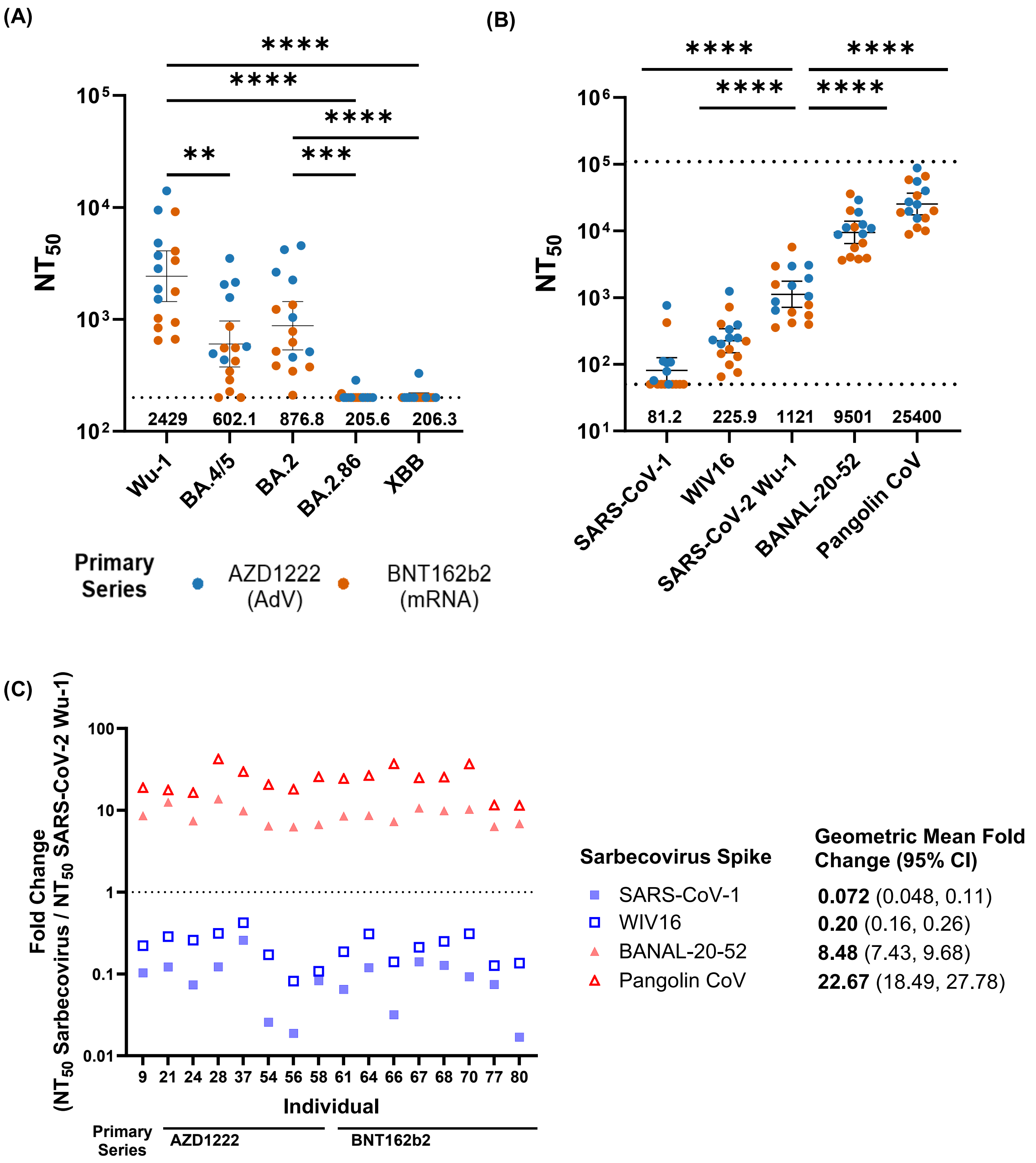
(A)
****
****
****
**
***
10^5
10^4
10^3
10^2
NT50
2429
602.1
876.8
205.6
206.3
Wu-1
BA.4/5
BA.2
BA.2.86
XBB
Primary Series
AZD1222 (AdV)
BNT162b2 (mRNA)
(B)
****
****
****
****
10^6
10^5
10^4
10^3
10^2
10^1
NT50
81.2
225.9
1121
9501
25400
SARS-CoV-1
WIV16
SARS-CoV-2 Wu-1
BANAL-20-52
Pangolin CoV
(C)
Fold Change
(NT50 Sarbecovirus / NT50 SARS-CoV-2 Wu-1)
100
10
1
0.1
0.01
9 21 24 28 37 54 56 58 61 64 66 67 68 70 77 80
Individual
Primary Series
AZD1222
BNT162b2
Sarbecovirus Spike
SARS-CoV-1
WIV16
BANAL-20-52
Pangolin CoV
Geometric Mean Fold Change (95% CI)
0.072 (0.048, 0.11)
0.20 (0.16, 0.26)
8.48 (7.43, 9.68)
22.67 (18.49, 27.78)

Supplementary Figure 4: Neutralisation of spikes from SARS-CoV-2 Wu-1, early omicron lineages, and related sarbecoviruses by sera from individuals receiving three vaccine doses. (A) Neutralisation of SARS-CoV-2 spike-pseudotyped lentiviruses, including SARS-CoV-2 Wu-1 D614G and early Omicron lineages (BA.4/5, BA.2.86, XBB) by sera collected 1 month after a third vaccine dose (n = 16) in HeLa-ACE2 cells. All individuals received mRNA vaccines for their third dose. 50% neutralising titres (NT50) shown as individual points (blue = primary two-dose series with AZD1222, orange = primary two-dose series with BNT162b2), with geometric mean titres (GMT) ± 95% CI overlaid. Dotted lines indicate min/max assay detection limits. P-values calculated using the Friedman test with Dunn's Multiple Comparisons correction. Data representative of two independent experiments, each with two technical replicates. (B) Neutralisation of sarbecovirus spike-pseudotyped lentiviruses (SARS-CoV-1, WIV16, BANAL-20-52, Pangolin CoV) by sera collected 1 month post-third dose (n = 16) in HEK293T-ACE2 cells. NT50 values presented as in (A). Statistical comparisons performed using the Wilcoxon matched-pairs signed-rank test relative to SARS-CoV-2 Wu-1 spike. Significance thresholds: *$p < 0.05$; **$p < 0.01$; ***$p < 0.001$; ****$p < 0.0001$; ns, not significant. Data representative of two independent experiments, each with technical replicates. (C) Fold change in neutralisation of spike-pseudotyped lentiviruses bearing sarbecovirus spikes relative to SARS-CoV-2 Wu-1 (dotted line at y = 1). Geometric mean fold change ± 95% CI summarized in the accompanying table.

Spike Protein	Accession ID	Host Species	Location	Date
SARS CoV-1 (HsZcc)	AY394995	*Homo sapiens*	Guangdong, China	2003
SARS-CoV-2 Wu-1 (D614G)	MN908947.3	*Homo sapiens*	Wuhan, China	2019
SARS-CoV-2 Alpha	EPI_ISL_3980577	*Homo sapiens*	Britain	2021
SARS-CoV-2 Delta	EPI_ISL_1635330	*Homo sapiens*	India	2021
SARS-CoV-2 Omicron BA.2	UJP23605.1	*Homo sapiens*	USA	2022
SARS-CoV-2 Omicron BA.4	UPP14409.1	*Homo sapiens*	USA	2022
SARS-CoV-2 Omicron BA.2.86	WOY09184.1	*Homo sapiens*	South Africa	2023
SARS-CoV-2 Omicron XBB	OP607807.1	*Homo sapiens*	USA	2022
P1E	EPI_ISL_410539	*Manis javanica*	Guanxi, China	2017
Pangolin CoV	EPI_ISL_410721	*Manis javanica*	Guangdong, China	2019
pCoV_GX-P4L	MT040333.1	*Manis javanica*	Guanxi, China	2017
RhGB01	MW719567.1	*Rhinolophus hipposideros*	Britain	2020
RShSTT200	EPI_ISL_852605	*Rhinolophus shameli*	Cambodia	2010
BANAL-20-103	MZ937001.1	*Rhinolophus pusillus*	Fueng, Laos	2020
BANAL-20-236	MZ937003.1	*Rhinolophus marshalli*	Fueng, Laos	2020
BANAL-20-52	MZ937000.1	*Rhinolophus malayanus*	Fueng, Laos	2020
Khosta-1	MZ190137.1	*Rhinolophus ferrumequinum*	Russia	2022
Khosta-2	MZ190138.1	*Rhinolophus hipposideros*	Russia	2022
RP3	DQ071615	*Rhinolophus pearsonii*	Guanxi, China	2004
Anlong-103	KY770858	*Rhinolophus sinicus*	Guizhou, China	2013
Longquan_140	KF294457	*Rhinolophus monoceros*	Guizhou, China	2012
HKU3-1	DQ022305	*Rhinolophus sinicus*	Hong Kong	2005
279_2005	DQ648857	*Rhinolophus macrotis*	Hubei, China	2004
Rc-o319	LC556375	*Rhinolophus cornutus*	Japan	2013
RpShaanxi 2011	JX993987	*Rhinolophus pusillus*	Shaanxi, China	2011
RacCS203	MW251308	*Rhinolophus acuminatus*	Thailand	2020
As6526	KY417142	*Aselliscus stoliczkanus*	Yunnan, China	2014
F46	KU973692	*Rhinolophus pusillus*	Yunnan, China	2012
JTMC15	KU182964	*Rhinolophus ferrumequinum*	Yunnan, China	2013
LYRa11	KF569996	*Rhinolophus affinis*	Yunnan, China	2011
RaTG13	EPI_ISL_402131	*Rhinolophus affinis*	Yunnan, China	2013
RaTG15	GWHBAUP01000001	*Rhinolophus affinis*	Yunnan, China	2015
RmYN02	EPI_ISL_412977	*Rhinolophus malayanus*	Yunnan, China	2019
RmYN05	MZ081376.1	*Rhinolophus malayanus*	Yunnan, China	2020
Rs4081	KY417143	*Rhinolophus sinicus*	Yunnan, China	2012
Rs4084	KY417144	*Rhinolophus sinicus*	Yunnan, China	2012
Rs4231	KY417146.1	*Rhinolophus sinicus*	Yunnan, China	2013
Rs4255	KY417149.1	*Rhinolophus sinicus*	Yunnan, China	2013
WIV-1	KF367457.1	*Rhinolophus sinicus*	Yunnan, China	2012
WIV-16	KT444582.1	*Rhinolophus sinicus*	Yunnan, China	2013
YN2016A	OK017847.1	*Rhinolophus sinicus*	Yunnan, China	2016
YN2020B	OK017852.1	*Rhinolophus sinicus*	Yunnan, China	2020
YN2020F	OK017856.1	*Rhinolophus sinicus*	Yunnan, China	2020
Yunnan 2011	JX993988	*Chaerephon plicata*	Yunnan, China	2011
ZC45	MG772933	*Rhinolophus sinicus*	Zheijang, China	2017

Supplementary Table 1: Accession IDs, host species and location/year of sampling for each sarbecovirus investigated in this study

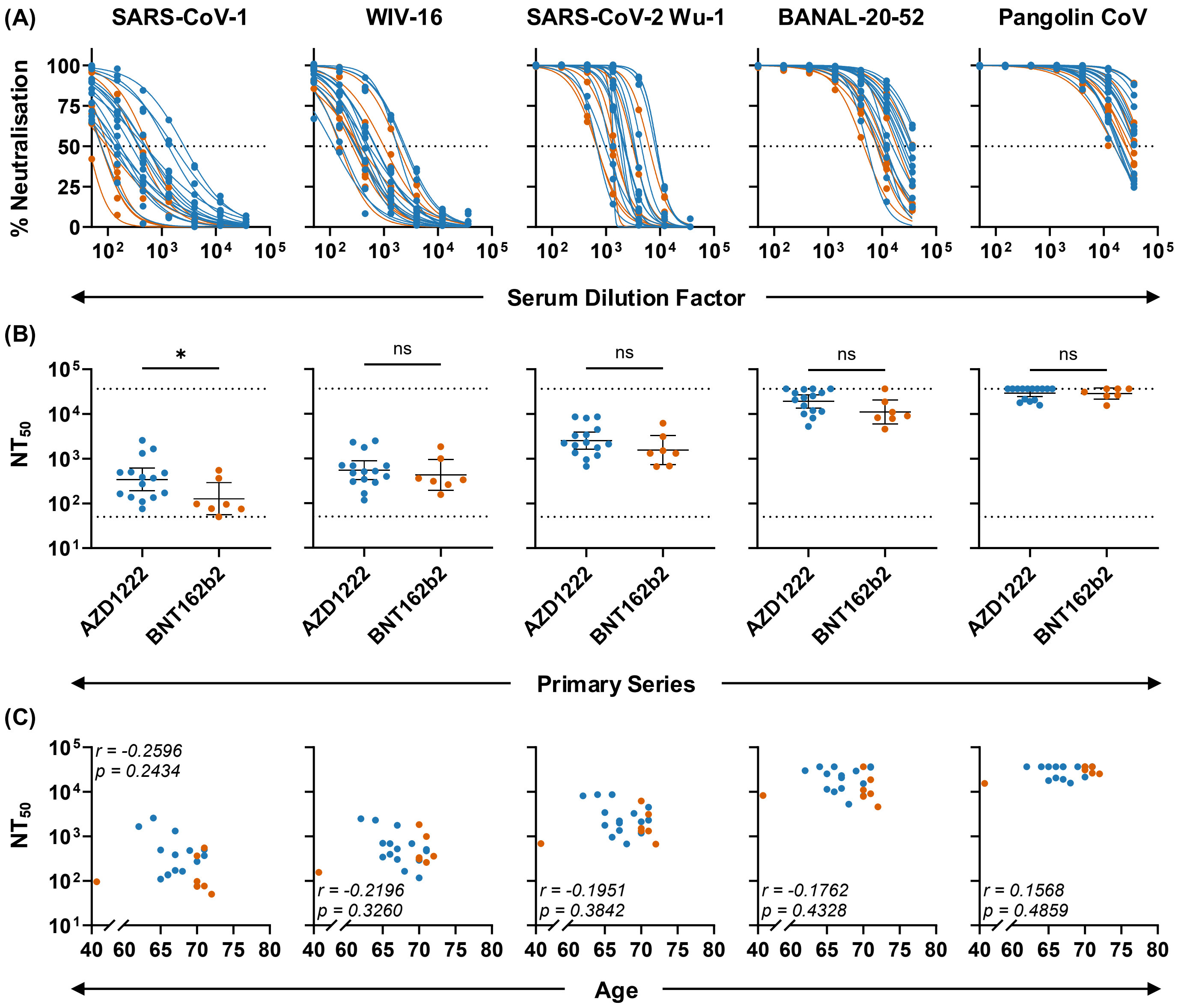

Supplementary Figure 5: Neutralisation curves and comparisons among age and primary series. (A) Neutralisation of sarbecovirus (SARS-CoV-1, WIV16, BANAL-20-52, Pangolin CoV) and SARS-CoV-2 Wu-1 spike-pseudotyped GFP lentiviruses after preincubation with n = 22 serum samples taken 1 month post-fourth dose in HEK293T cells stably expressing human ACE2. Values were normalised to cells-only and virus-only wells. Blue points indicate a primary two-dose series with AZD1222 and orange points indicate a primary two-dose series with BNT162b2. The dotted line at y = 50 indicates the point at which 50% of the pseudotyped virus was neutralised by each serum sample. (B) 50% neutralising titres (NT50) stratified by primary vaccine series (AZD1222 or BNT162b2) with bars indicating geometric mean titre (GMT) with 95% CI. P-values were calculated using a Mann Whitney test. (C) Correlation of age and NT50 against spike-pseudotyped lentiviruses. Spearman r values and p-values were calculated using the nonparametric Spearman correlation test. *$p < 0.05$; **$p < 0.01$; ***$p < 0.001$; ****$p < 0.0001$; ns, $p > 0.05$.

(A)

Primary 2-dose series	Male, %	Female, %	Age, years (median, IQR)
**AZD1222 (AdV)**	2 (20%)	3 (30%)	68 (67, 69)
**BNT162b2 (mRNA)**	3 (30%)	2 (20%)	72 (72, 72)
**Total**	**5 (50%)**	**5 (50%)**	**70.5 (68.25, 72)**

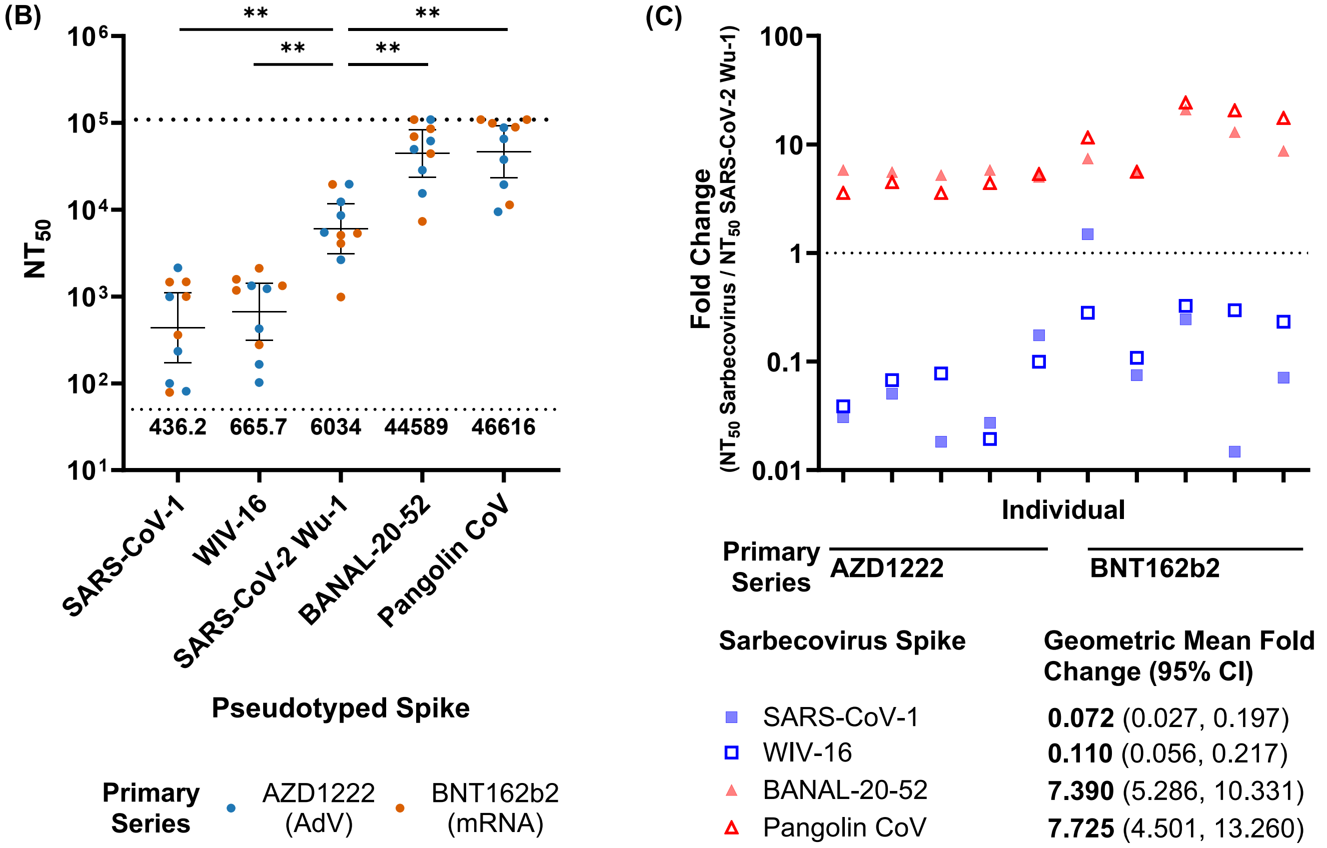

Supplementary Figure 6: Neutralisation of sarbecovirus spikes by sera from individuals receiving four vaccinations in second cohort (n = 10). (A) Second cohort characteristics. Age- and sex-matched individuals (n = 10) vaccinated four times against SARS-CoV-2. All individuals received a primary two-dose series of either AZD1222 (adenovirus vector-based) or BNT162b2 (mRNA-based) against SARS-CoV-2 Wuhan-Hu-1 (Wu-1), followed by a 3rd dose (mRNA-based) against Wu-1 and a fourth bivalent dose (mRNA-based) against Wu-1 and B.1.1.529 (Omicron BA.1). (B) GFP neutralisation assay of sarbecovirus spike-pseudotyped lentiviruses after preincubation with n = 10 serum samples taken 1 month post-fourth dose in HEK293T cells stably expressing human ACE2. 50% neutralising titres (NT50) of cohort sera against spike-pseudotyped lentiviruses are shown with individual points (blue = primary two-dose series with AZD1222, orange = primary two-dose series with BNT162b2) and bars indicating geometric mean titre (GMT) with 95% CI. Dotted lines indicate the minimum and maximum detection limits of the neutralisation assay. GMT is written below the minimum detection limit dotted line. Representative graph of n = 2 independent experiments. P-values were calculated using the Wilcoxon matched-pairs signed rank test relative to Wu-1. *$p < 0.05$; **$p < 0.01$; ***$p < 0.001$; ****$p < 0.0001$; ns, $p > 0.05$. (C) Fold change in neutralisation of lentiviruses pseudotyped with sarbecovirus spikes compared with SARS-CoV-2 Wu-1 (dotted line at y = 1) for each individual. Geometric mean fold change and 95% CI were calculated to compare neutralisation of each pseudotyped sarbecovirus spike to SARS-CoV-2 Wu-1. Representative graph of n = 2 independent experiments.

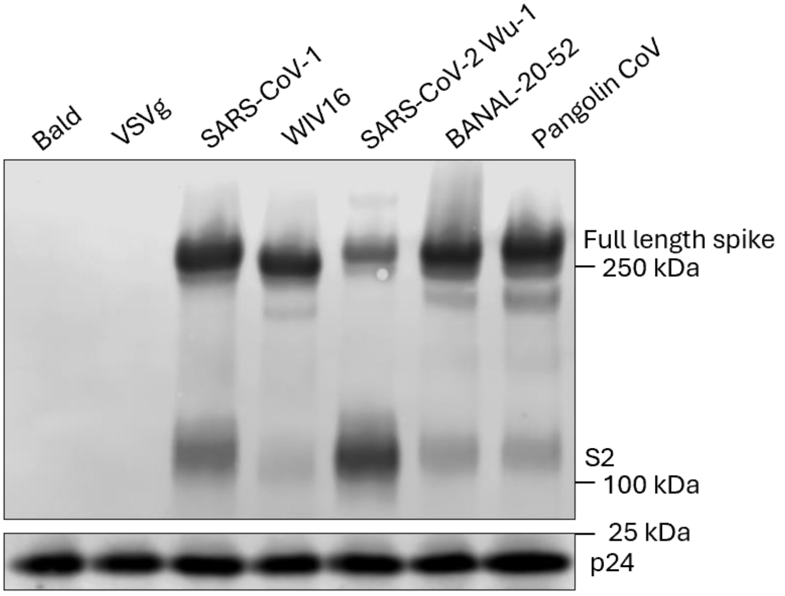

Spike	Total expression (relative to SARS-CoV-2 Wu-1)
SARS-CoV-1	1.2115
WIV-16	0.8797
SARS-CoV-2 Wu-1	1.0000
BANAL-20-52	1.0535
Pangolin CoV	1.0715

Supplementary Figure 7: Western blot analysis comparing spike incorporation. Left to right: bald pseudotyped virus with no entry receptor, pseudotyped virus with VSV glycoprotein, SARS-CoV-1 spike, WIV-16 spike, SARS-CoV-2 spike, BANAL-20-52 spike, pangolin CoV spike. Uncleaved full length spike was around 260 kDa, cleaved S2 subunit was around 100 kDa, p24 loading control is 24 kDa.

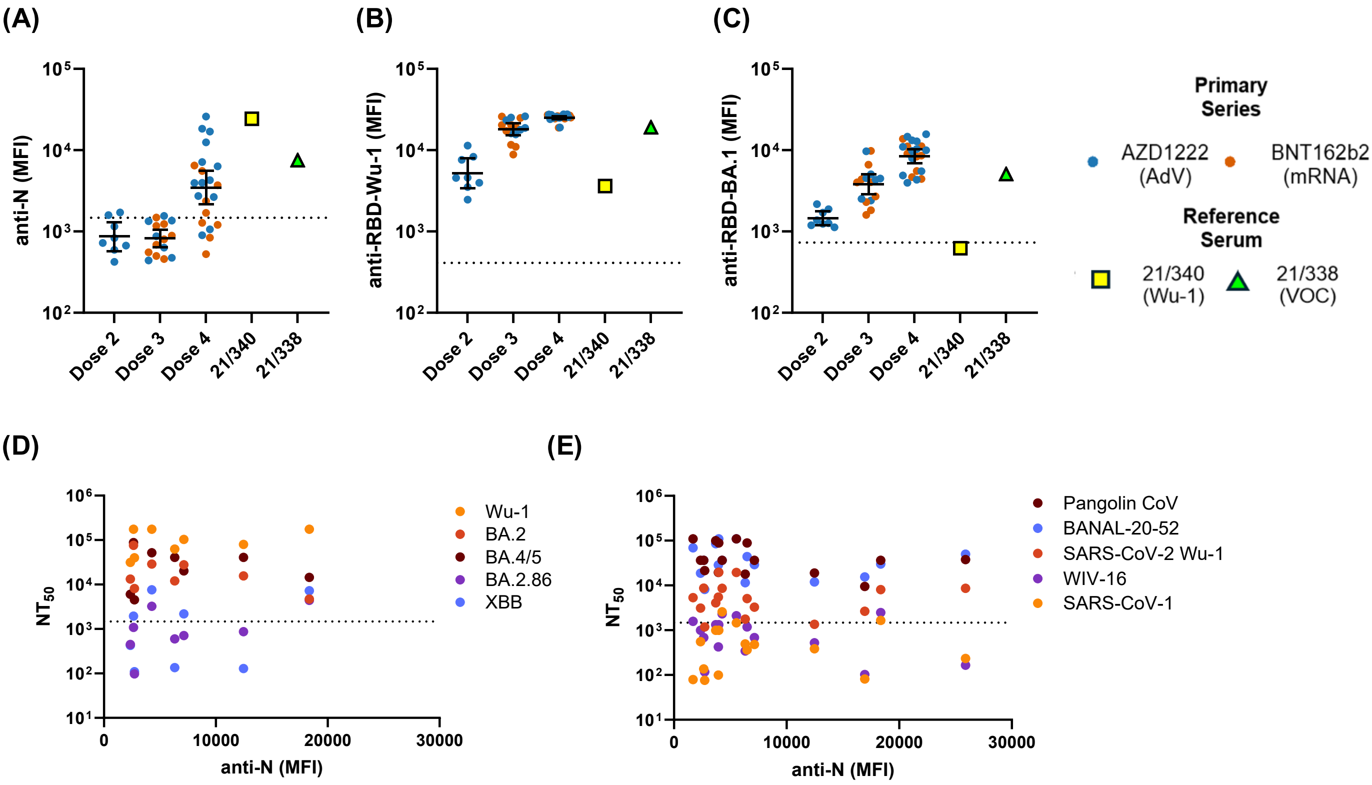

Supplementary Figure 8: Serum IgG binding to SARS-CoV-2 antigens in a sample of individuals from our previous two cohorts Binding to (A) SARS-CoV-2 Wu-1 nucleocapsid (N), (B) Wu-1 RBD, and (C) Omicron BA.1 RBD measured in samples collected post-dose 2 (n = 8), 3 (n = 16), and 4 (n = 22). Individuals receiving a primary two-dose series with AZD1222 are shown in blue, while those receiving BNT162b2 are shown in orange. Dotted lines represent cutoffs for seropositivity determined by the mean + 3 SD in n = 93 pre-pandemic healthy donor samples. Cutoffs are 1473.8 (anti-N), 411.91 (anti-RBD-Wu-1), and 729.1 (anti-RBD-BA.1). Reference NIBSC WHO serum included for comparison: Yellow square = 21/340 (exposure to ancestral strain only) and green triangle = 21/338 (Vaccination combined with exposure to pre-omicron VOC). Bars represent geometric mean ± 95% CI. Comparison between NT50 and serum anti-N binding are shown for (D) Omicron variants of concern and (E) sarbecoviruses.

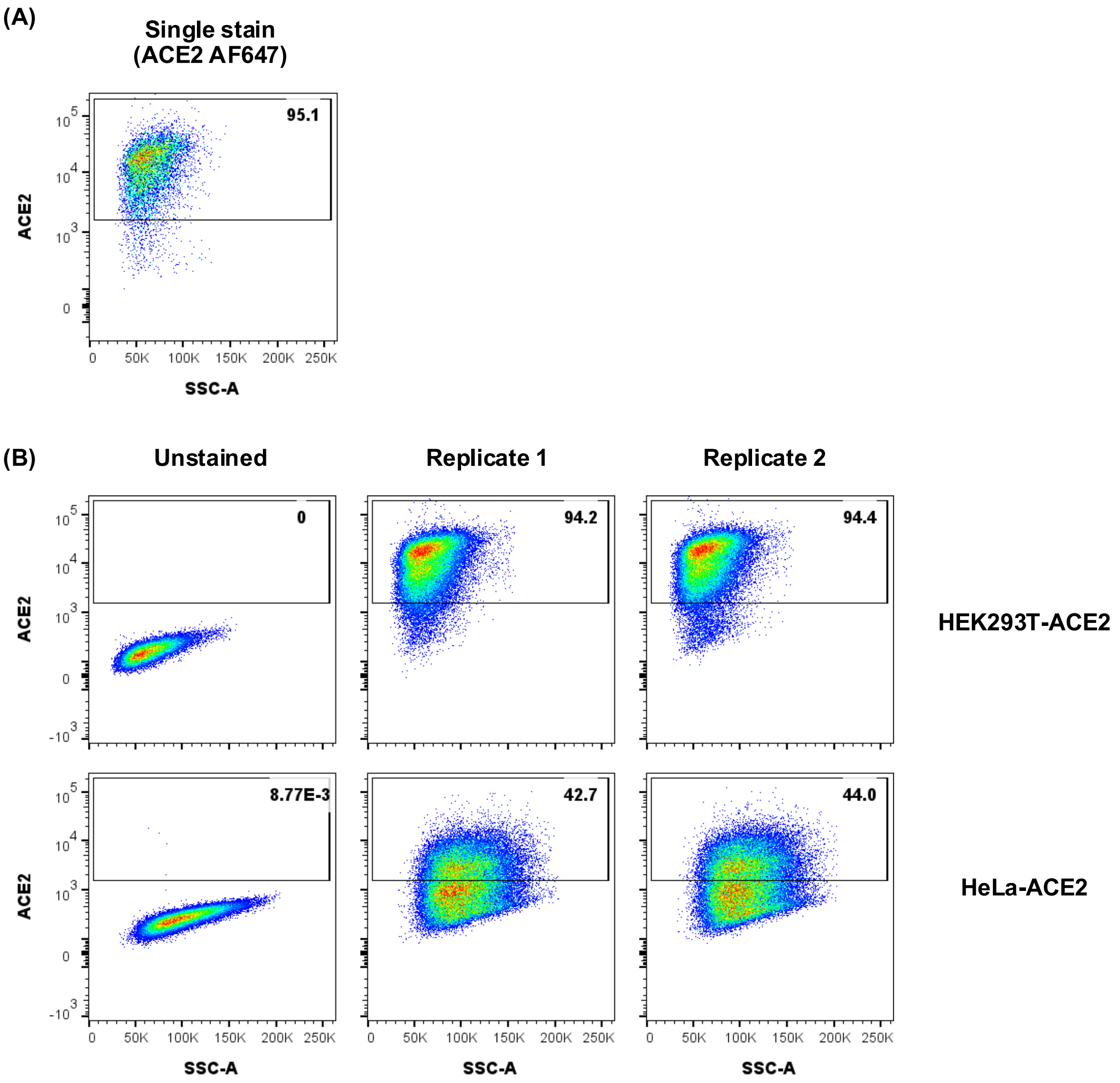

Supplementary Figure 9: ACE2 expression in HEK293T-ACE2 and HeLa-ACE2 cells. (A) Alexa Fluor 647 (ACE2) single stain to determine positive population (B) HEK293T-ACE2 (top) and HeLa-ACE2 (bottom) cells were stained in duplicate and gated on live single cells to confirm expression of ACE2 (Alexa Fluor 647, y-axis). Left, unstained; middle, replicate 1; right, replicate 2.

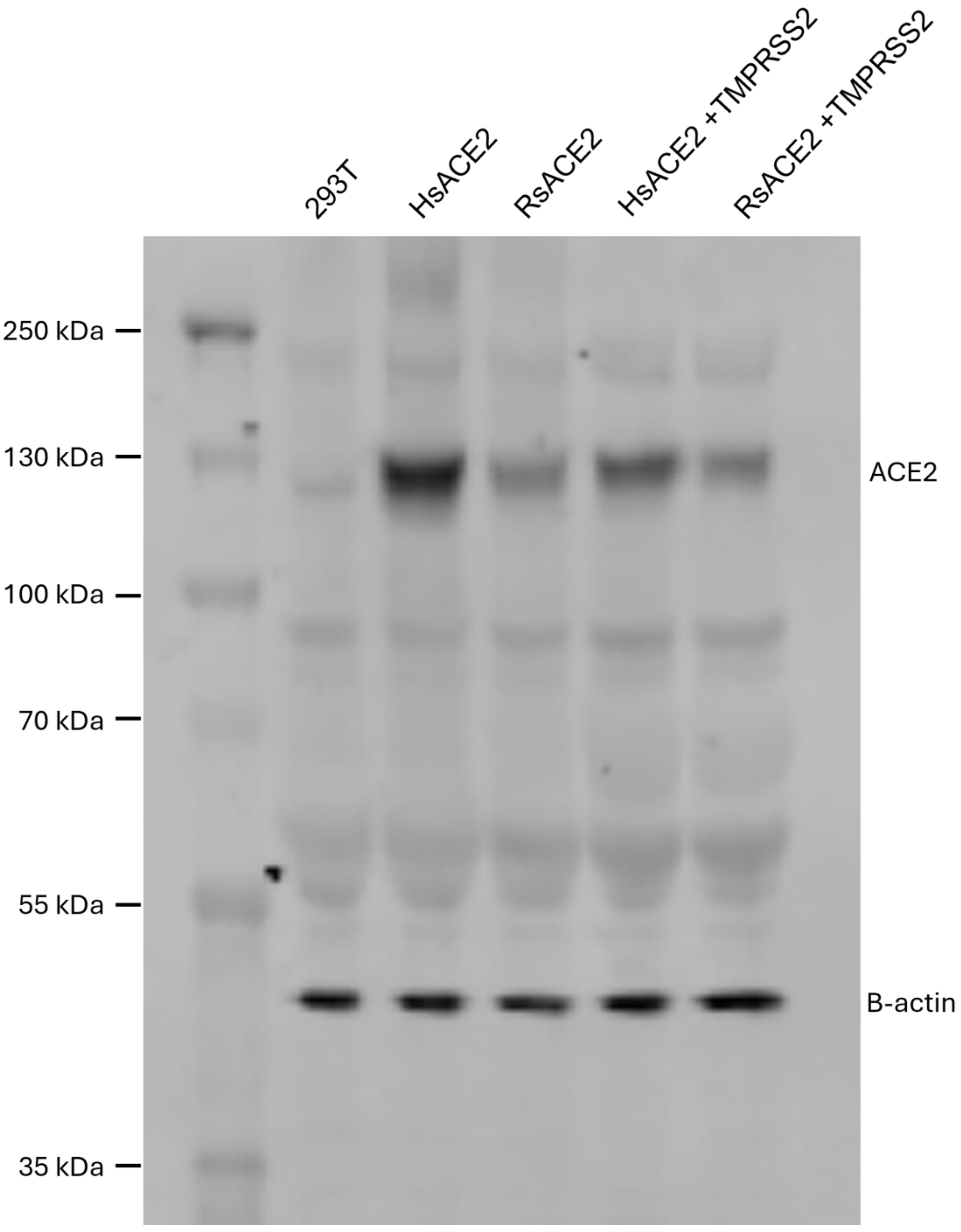

Supplementary Figure 10: Uncropped western blot of ACE2 and B actin

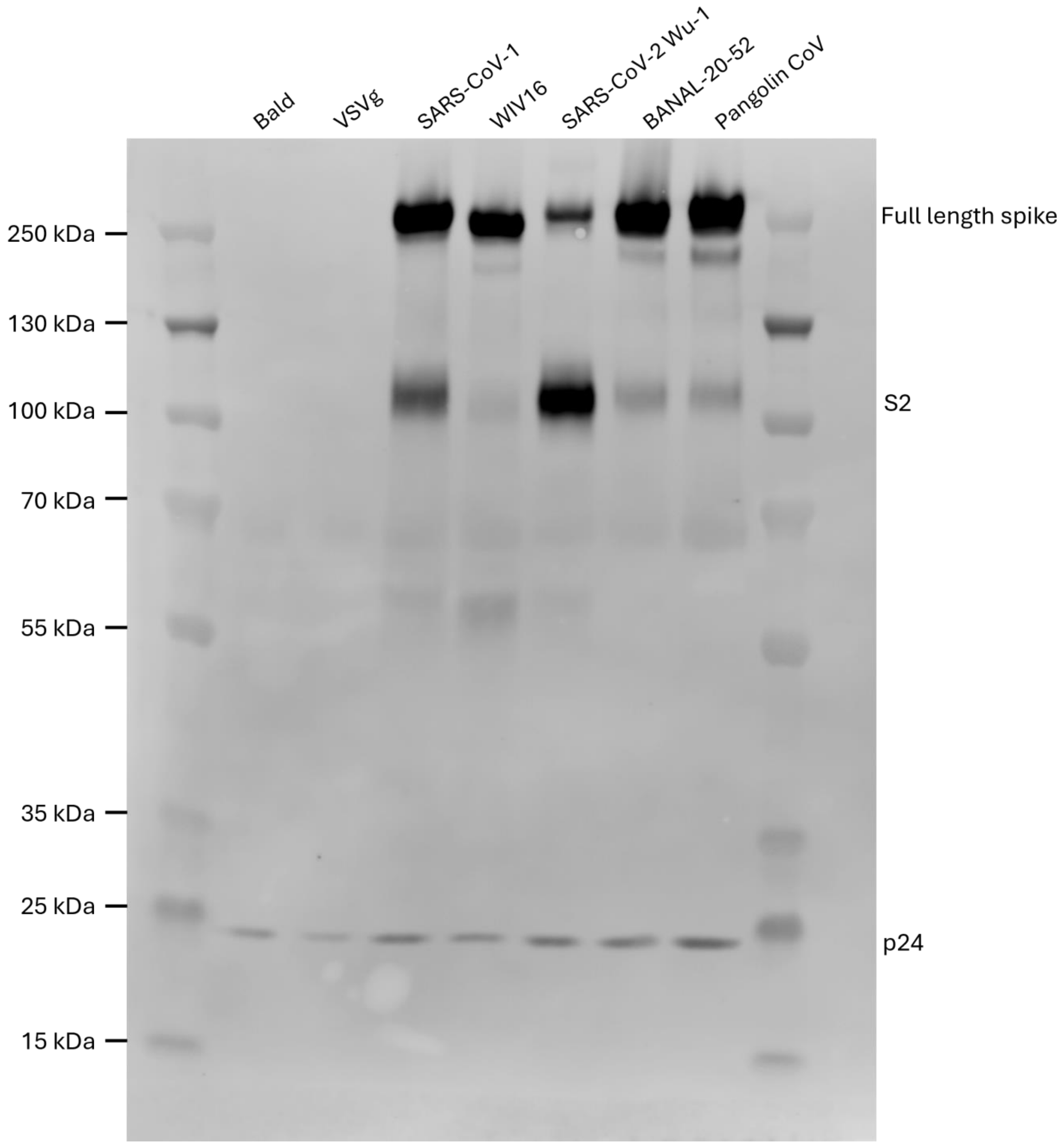

Supplementary Figure 11: Uncropped western blot of Spike and HIV-1 p24

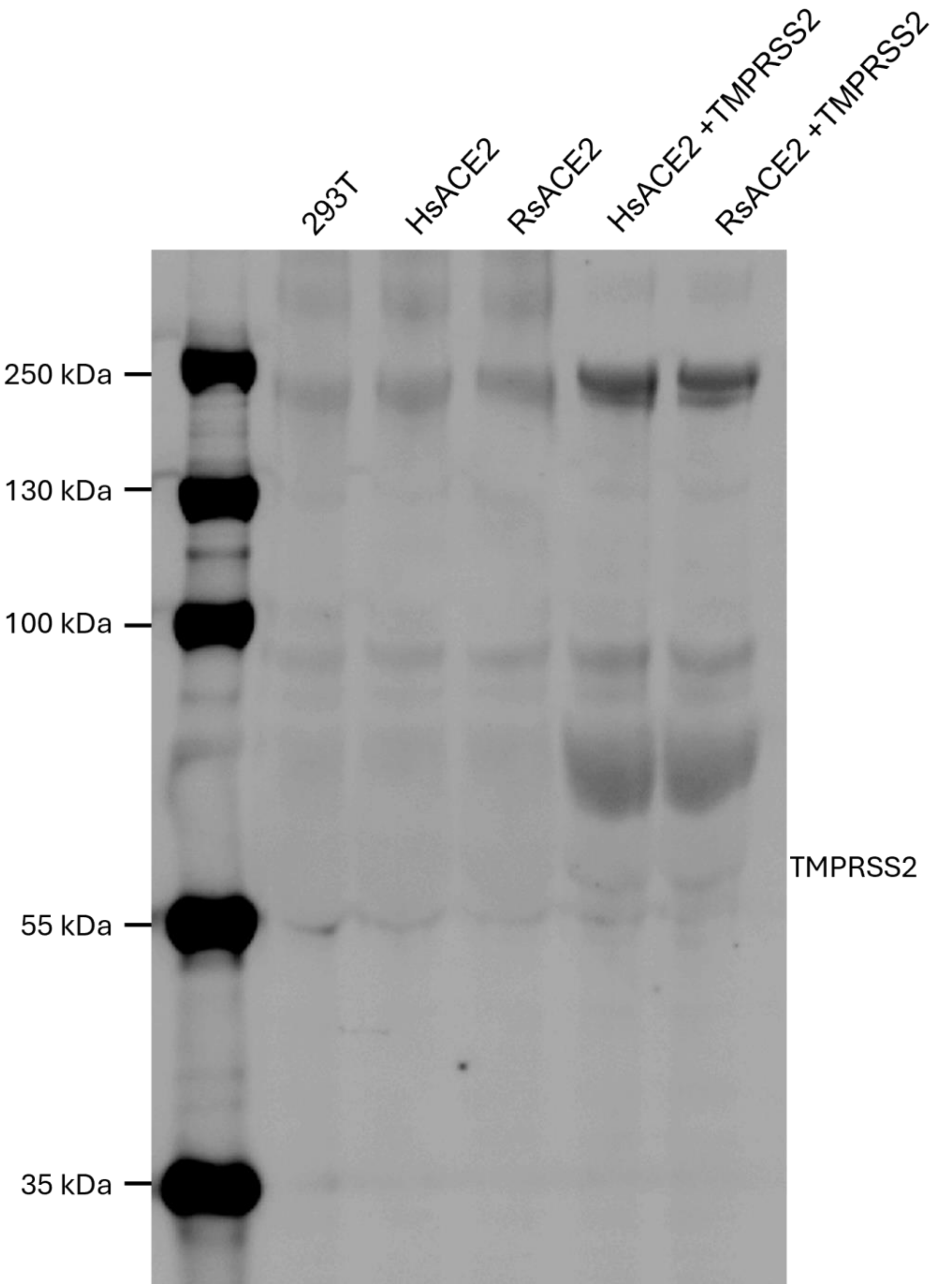

Supplementary Figure 12: Uncropped western blot of TMPRSS2
